# Supplementary material for: 15-Lipoxygenase promotes resolution of inflammation in lymphedema by controlling Treg cell function through IFN-β
Source: Nat Commun. 2024 Jan 4;15:221. doi: 10.1038/s41467-023-43554-y (PMC10766617; doi:10.1038/s41467-023-43554-y)
Supplement: Supplementary file 1 — Supplementary Information [file 41467_2023_43554_MOESM1_ESM.pdf]

SUPPLEMENTARY INFORMATION

Supplementary Figure 1

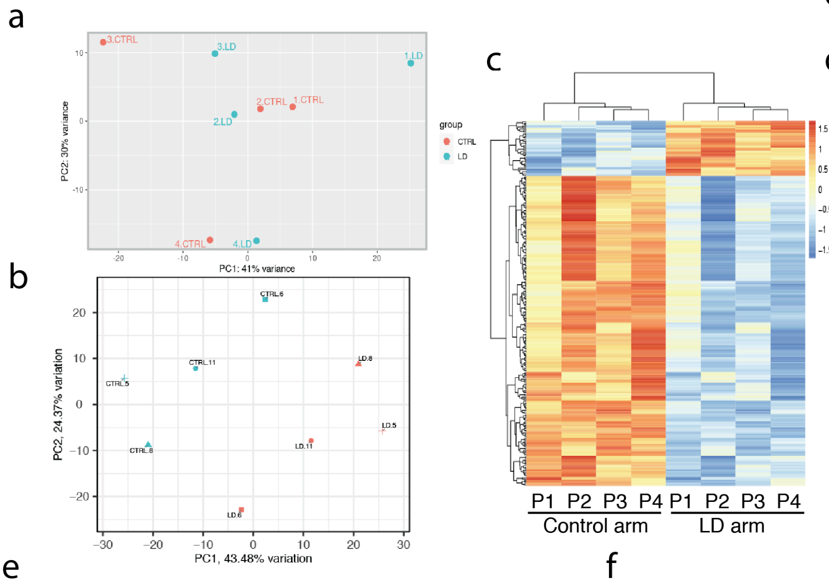

d

gene	log2(fold_change)	p_value
FCGBP	-1.404	****
IGSF21	-1.2154	*
HPGDS	-1.0974	**
CD24	-0.9216	*
TPSB2	-0.9196	**
CTSG	-0.8104	**
CPA3	-0.7376	**
MS4A2	-0.7198	*
CD9	-0.7166	**
CCL13	-0.6891	*
PPBP	-0.6141	*
MS4A6A	-0.5495	*
TGFBI	-0.586227	*
CAPG	-0.52	*

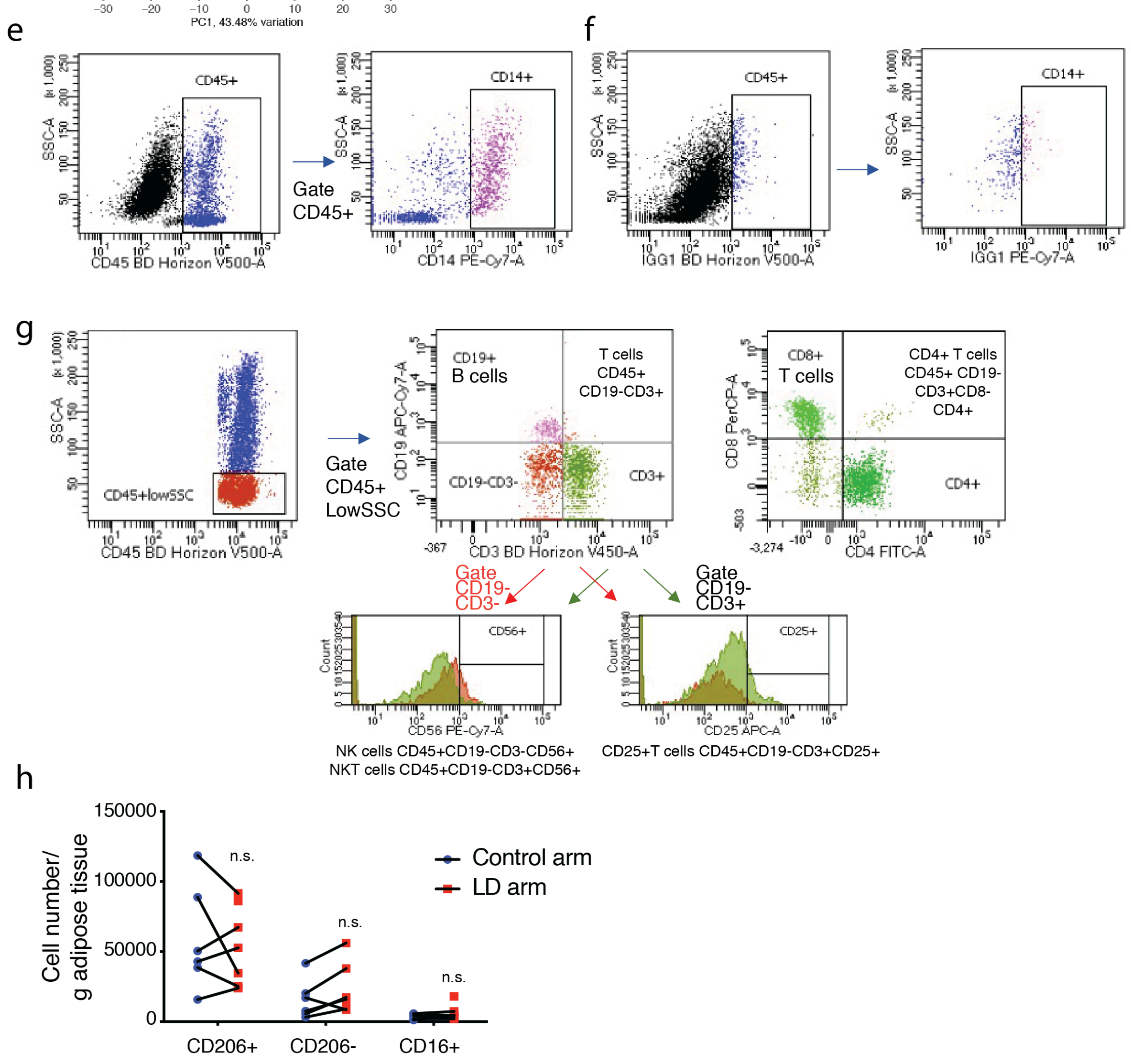

Supplementary Figure 1: Human LD RNAseq expression profile

a. Principle component analysis (PCA) plot for RNA seq data of biological replicates of biopsies from control arm (CTRL) and lymphedema arm (LD) on each patient. **b**. PCA plot realized after the batch effect correction showing clear separation by condition (CTRL versus LD). **c**, Hierarchical clustering heatmap of the differentially expressed genes between the CTRL and the LD condition of the RNA-seq data, after the batch effect of the individual n.1 was corrected. **d**, Downregulated genes associated with immune response. **e-g**. Representative dot plots of the flow cytometry analyses are shown starting from viable cells gated on side scatter (SSC)/forward scatter (FSC) of the stroma vascular cells (not shown). **e**. Macrophages, defined as CD45+/CD14+ are gated from CD45+ cells from the CD45/SSC dot plot, **f**. positioned from dot plots obtained with respective isotype controls. **g**. T lymphocytes, defined as CD45+/CD19-/CD3+ and B lymphocytes defined as CD45+/CD3-/CD19+ are gated from CD45+/low SCC. T lymphocytes are further divided into CD4+ T lymphocytes and CD8+ T lymphocytes. NK (CD3-/CD56+) and NKT (CD3+/CD56+) are gated from CD19-/CD3- cells and CD19-/CD3+ cells respectively and CD25+ T cells from CD19-/CD3+ cells. **h**. Flow cytometry analysis of CD206+ and CD16+ cells in LD dermolipectomies.

For **a-d**, *n*=4 women with LD (tissue biopsies from normal arm and LD arm from the same patient). For **e-h**, *n*=6 women with LD (tissue biopsies from normal arm and LD arm from the same patient). (**e**) Wald test was used to generate p-values and log2 fold changes. (**h**) *P* values are derived from two-way ANOVA. Source data are provided as a Source data file.

Supplementary Figure 2

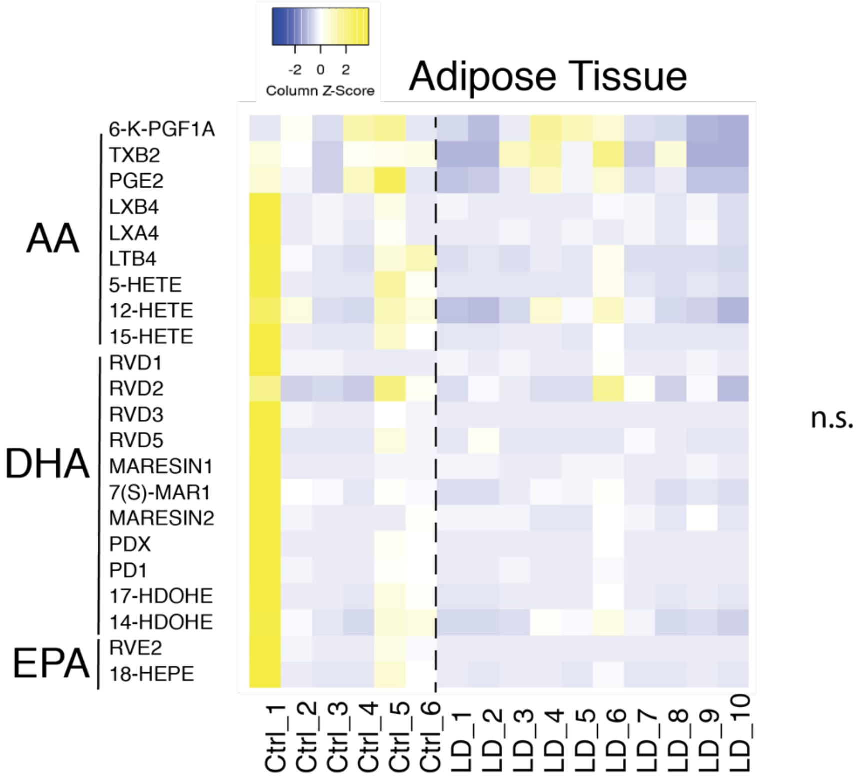

Supplementary Figure 2: Human LD lipidomic analysis

Heatmap of lipid mediators derived from arachidonic acid (AA), Docosahexaenoic acid (DHA) and Eicosapentaenoic acid (EPA) analysis in lymphedematous adipose tissue from women who developed LD after breast cancer. *n*=4 women with LD (tissue biopsies from normal arm and LD arm from the same patient) and n=6 women with LD (LD arm tissue biopsies only). Source data are provided as Supplementary tables 3-5.

Supplementary Figure 3

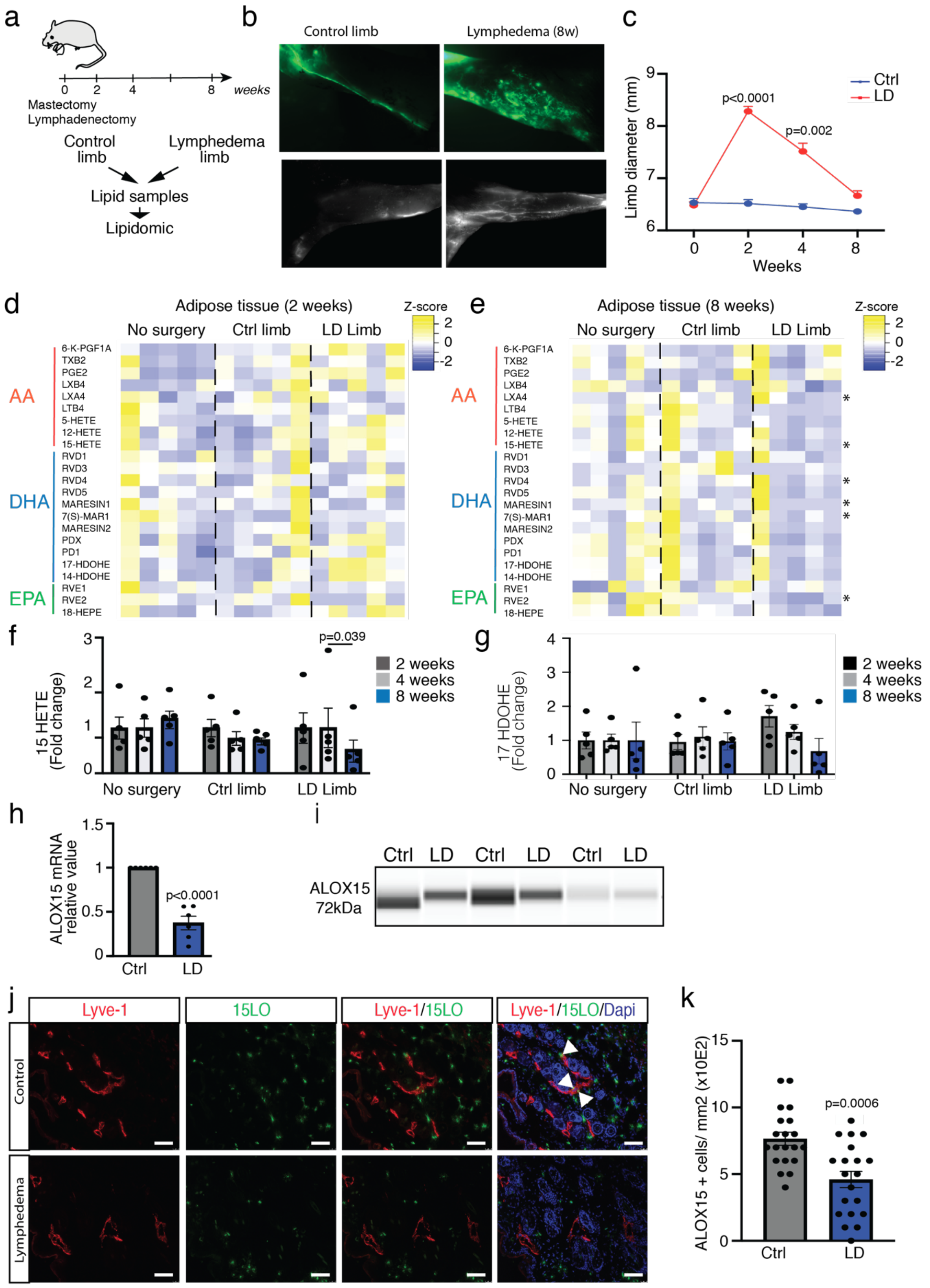

Supplementary Figure 3: Downregulation of ALOX15 in a mouse model of lymphedema

a, Schematic representation of the experimental procedure. **b**, Lymphography of the limb from mice with LD. **c**, Quantification of the limb diameter in mice with LD. d-e, Heatmap of lipid mediators derived from arachidonic acid (AA), Docosahexaenoic acid (DHA) and Eicosapentaenoic acid (EPA) analysis in lymphedematous adipose tissue from mice 2 weeks (**d**) and 8 weeks (**e**) post-surgery. **f**, 15-HETE dosage in mouse LD (*P<0.05). **g**, Quantification of 17-HDOHE in mouse LD. **h**, *Alox15* mRNA expression in mouse LD. **i**, 15-LO protein expression in mouse LD. **j**, Immunodetection of 15-LO (green) and LYVE-1 (red) in lymphedematous skin (Scale bar: 50µm). **k**, Quantification of the 15-LO-positive cells in mouse LD (***P<0.001). For **a-d**, *n*=4 women with LD (tissue biopsies from normal arm and LD arm from the same patient). For **b,c** *n*=6 mice per group. For **d,g** *n*=5 mice per group. For **h** *n*=6-7 mice per group. For **i** *n*=3 mice. For **j,k** *n*=4 mice per group. Data are shown as mean ± s.e.m. (**f,g**) *P* values are derived from two-way ANOVA. (**d,e)** *P* values are derived from unpaired t-test. (**f,g**) *P* values are derived from one-way ANOVA. (**k)** *P* values are derived from unpaired t-test. Source data are provided as a Source data file.

Supplementary Figure 4

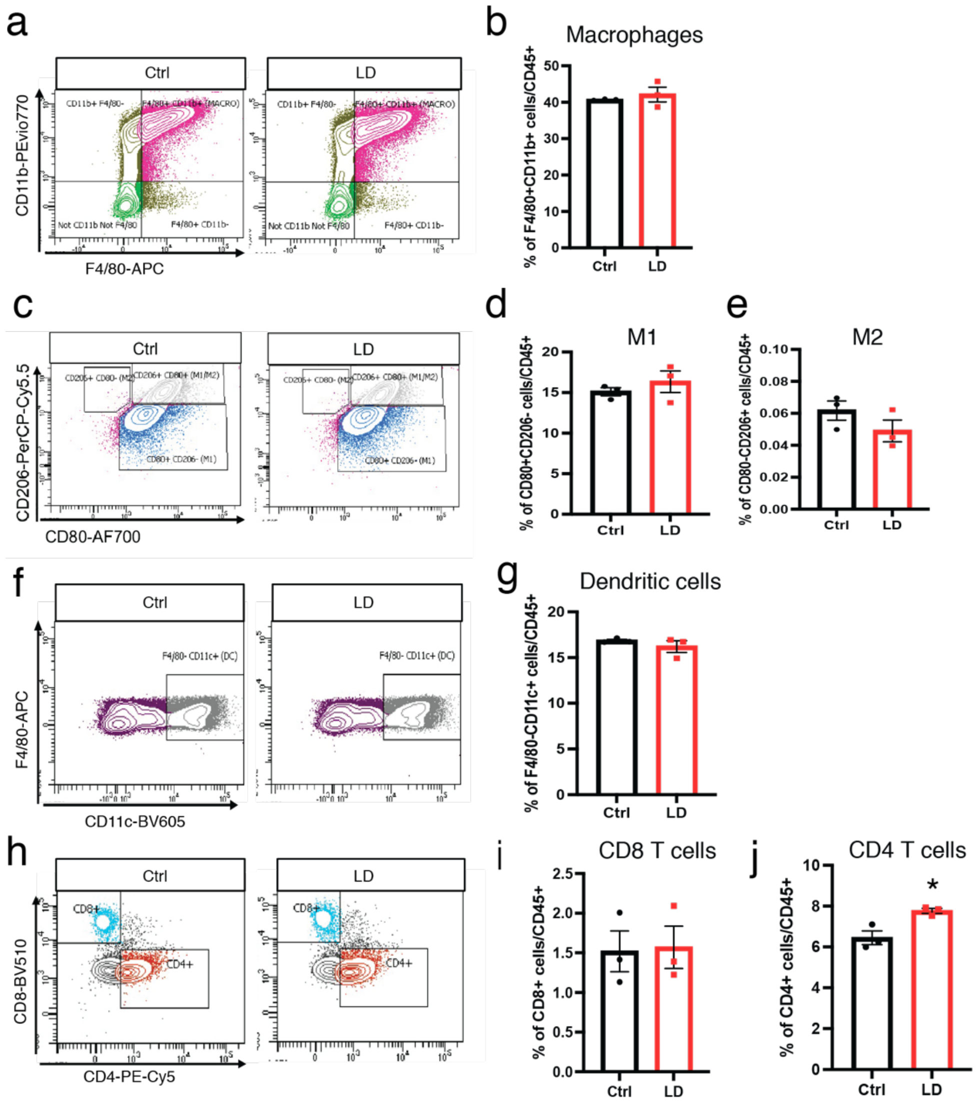

Supplementary Figure 4: Flow cytometry analysis of AT samples in mice LD

a, Representative FACS plots of LD macrophages. Gating method for the identification of macrophages (F4/80+/CD11b+). **b**, Quantification showing the proportion of macrophages in Ctrl and LD AT. **c**, Representative FACS plots of LD M1 and M2 macrophages. Gating method for the identification of macrophages (CD80+/CD206+). **d**, Quantification showing the proportion of M1 and M2 macrophages in Ctrl and LD AT. **e**, Representative FACS plots of LD dendritic cells. Gating method for the identification of dendritic cells (CD11c+/F4/80-). **f**, Representative FACS plots of LD dendritic cells. **g**, Quantification showing the proportion of dendritic cells in Ctrl and LD AT. **h**, Representative FACS plots of LD lymphocytes. Gating method for the identification of TCD4+ and TCD8+ cells. **i**, Quantification showing the proportion of CD8+ T cells in Ctrl and LD AT. **j**, Quantification showing the proportion of CD4+ T cells in Ctrl and LD AT. For **a-j,** *n*=3 mice per group. (**b,d,e,g,I,j**) *P* values are derived from unpaired t-test. Source data are provided as a Source data file.

Supplementary Figure 5

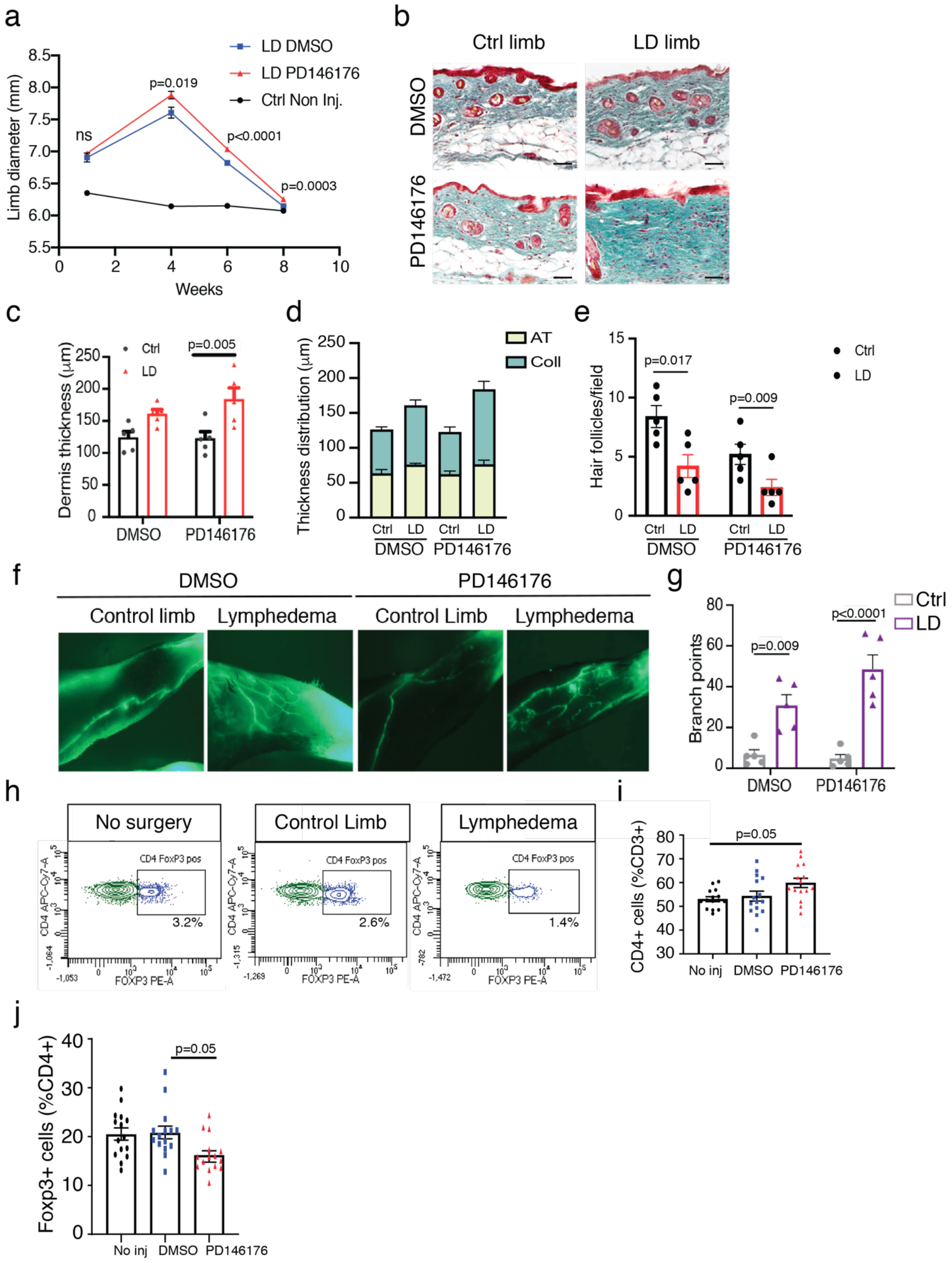

Supplementary Figure 5: ALOX15 inhibitors reduce Treg number in LD

a, Quantification of the limb diameter in mice with LD treated with 15-LO inhibitor (PD146176). **b**, Masson's trichrome coloration of the lymphedematous skin. **c**, Quantification of dermis thickness in mice with LD treated with PD146176. **d**, Skin thickness repartition related to collagen (Coll) vs adipose tissue (AT). **e**, Hair follicle quantification in LD skin. **f**, Lymphography of the limb from mice with LD treated with PD146176. **g**, Quantification of lymphatic branch point in the limb from mice with LD treated with PD146176. **h**, Representative FACS plots of LD CD4+ lymphocytes and Treg cells in LD. **i-j**, Flow cytometry analysis of CD4+ (**i**) and Foxp3+ (**j**) cell populations in spleen from mice treated with PD146176. For **a** n=10 mice per group. For **b-j** n=5 mice per group. Data are shown as mean ± s.e.m. (**a,c,e,g**) P values are derived from two-way ANOVA. **(i,j)** P values are derived from one-way ANOVA. Source data are provided as a Source data file.

Supplementary Figure 6

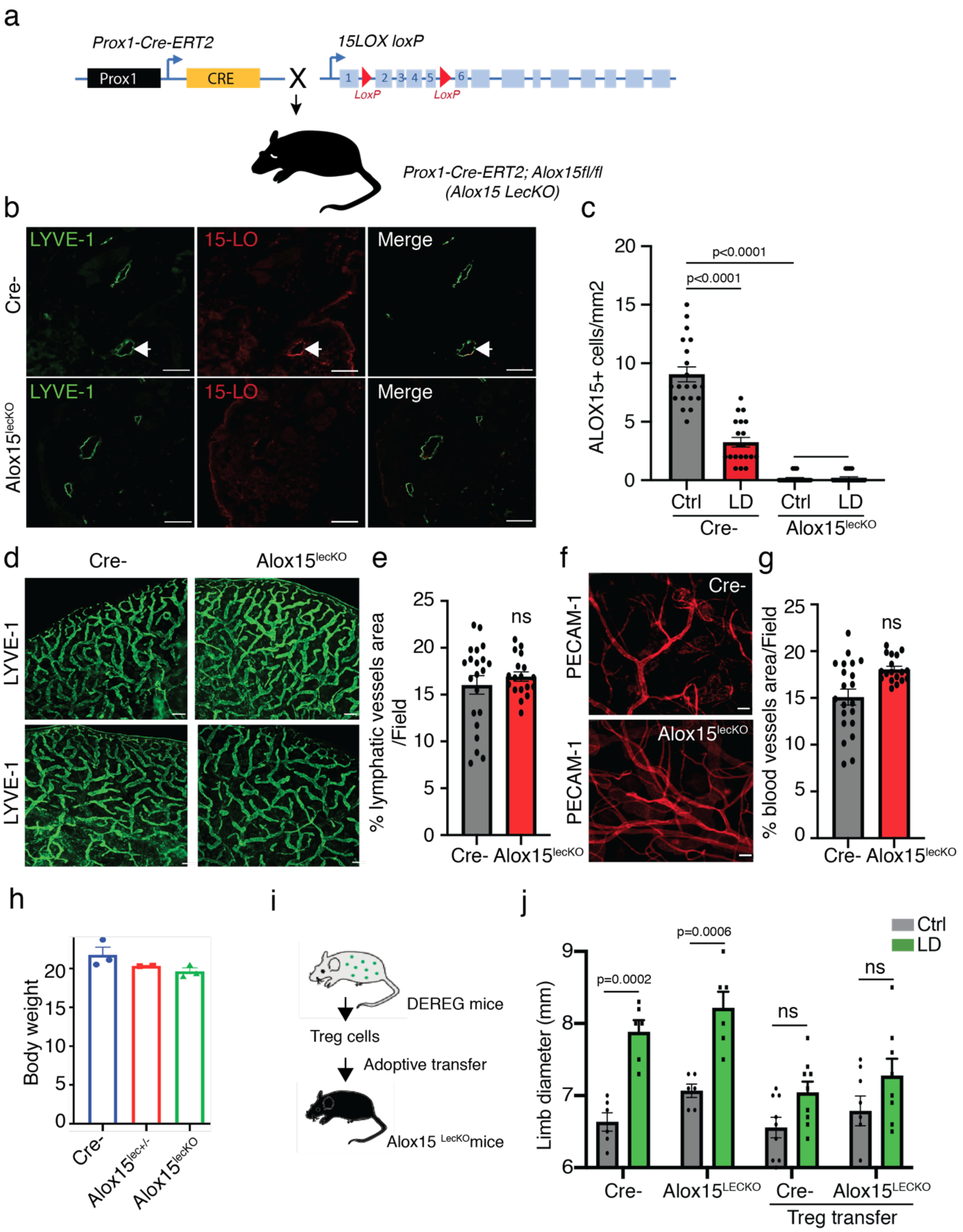

Supplementary Figure 6: ALOX15 lymphatic knock down had no effect on basal vascular networks.

a, Schematic representation of ALOX15^{LECKO} transgenic mice. **b**, Immunodetection of 15-LO (red) and Lyve-1 (green) in ALOX15^{LECKO} and Cre- control littermates' skin (Scale bar: 50µm). **c**, Quantification of ALOX15-positive LEC in mice's skin. **d**, Lyve-1 immunodetection of the lymphatic dermal network in ALOX15^{LECKO} mice (Scale bar: 25µm). **e**, Quantification of dermal lymphatic vessel density in ALOX15^{LECKO} mice. **f**, PECAM-1 immunodetection of the blood vessels in ALOX15^{LECKO} mice (Scale bar: 25µm). **g**, Quantification of dermal blood vessel density in ALOX15^{LECKO} mice. **h**, ALOX15 knock-down in the lymphatic system has no effect on body weight. **i**, Schematic representation of Treg cells adoptive transfer from DEREG mice to ALOX15^{LECKO} mice. **j**, Quantification of the limb diameter in ALOX15^{LECKO} mice with LD after Treg transfer in the limb AT. For **b-g** n=10 mice per group. For **h** n=3 mice per group. For **j** n=6-9 mice per group. Data are shown as mean ± s.e.m. (**c,j**) P values are derived from two-way ANOVA. (**h)** P values are derived from one-way ANOVA. (**e,g**) P values are derived from unpaired t-test. Source data are provided as a Source data file.

Supplementary Figure 7

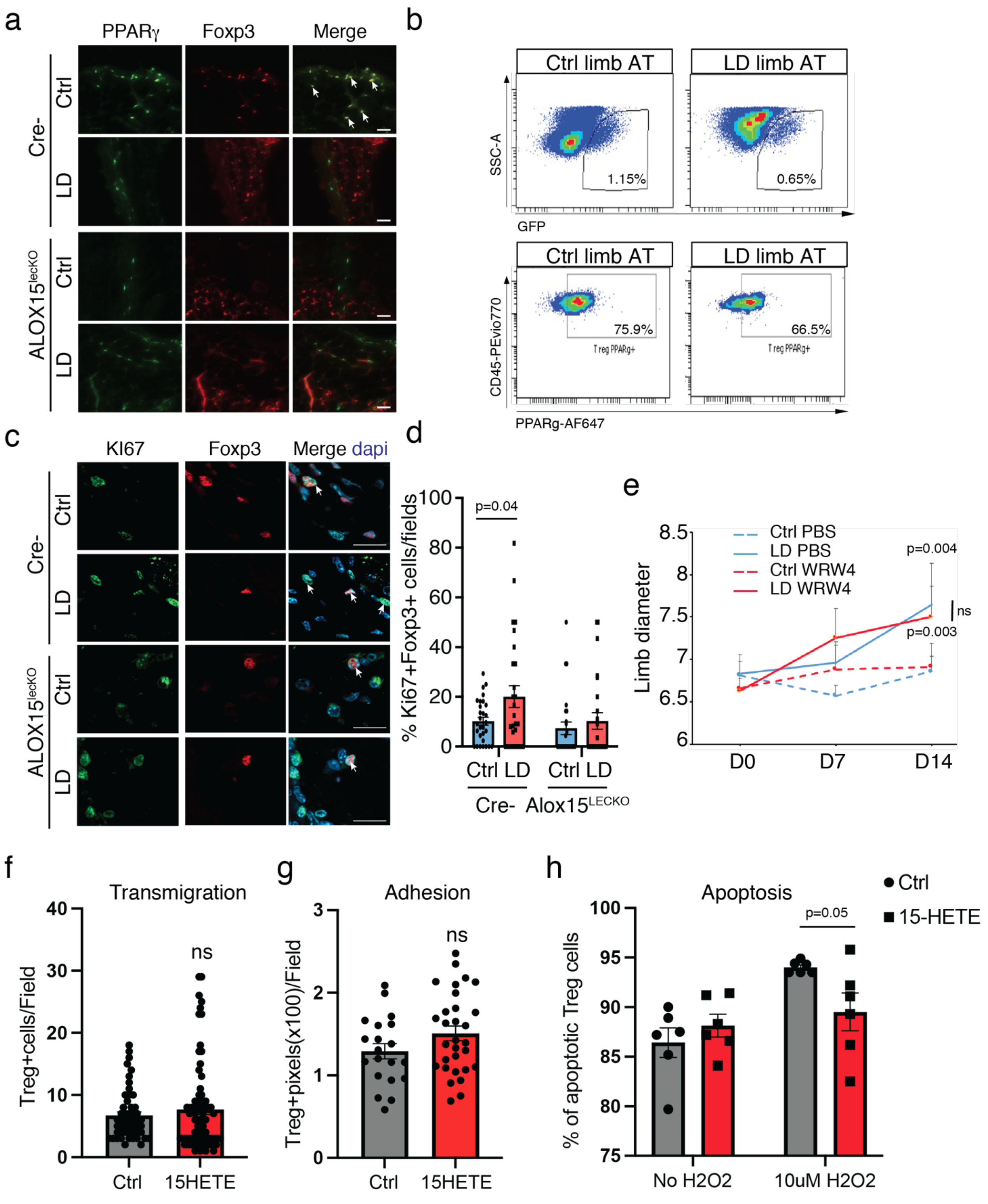

Supplementary Figure 7: Lymphatic endothelium protects Treg cells from apoptosis

a, Immunodetection of PPARg (green) and Foxp3 (red) in ALOX15^{LECKO} and Cre- control littermates' skin (Scale bar: 50µm). **b**, Representative FACS plots of AT from Ctrl and LD Treg cells (GFP+) and PPARg+ cells. Gating method for the identification of PPARg+ Treg cells. **c**, Immunodetection of proliferative Treg cells using KI67 (green) and Foxp3 (red) in ALOX15^{LECKO} and Cre- control littermates' skin (Scale bar: 50µm). **d**, Quantification of the percentage of KI67-positive Treg cells. **e**, Quantification of the limb diameter in ALOX15^{LECKO} mice with LD treated with WRW4 inhibitor. **f**, Quantification of Treg trans-lymphatic endothelial migration. **g**, Quantification of Treg cells adhesion to the lymphatic endothelial monolayer. **h**, Quantification of apoptotic Treg cells treated with 15-HETE.

For **a-d** *n*=5 mice per group. For **e** *n*=7 mice per group. For **f-f** *n*=3 independent replicates. Data are shown as mean ± s.e.m. (**d**) *P* values are derived from multiple Mann-Whitney analysis. (**e**) *P* values are derived from two-way ANOVA. (**f-h**) *P* values are derived from unpaired t-test. Source data are provided as a Source data file.

	Log2 FC	-log10(padj)		Log2 FC	-log10(padj)		Log2 FC	-log10(padj)		Log2 FC	-log10(padj)		Log2 FC	-log10(padj)
CETP	1,757	25,9	SOX17	0,729	2,72	OASL	0,64	1,85	LINC00639	0,569	1,84	PTPRT	-0,734	2,17
ADAMTSL1	1,135	6,71	IGSF10	0,723	2,57	MYH2	0,639	1,35	RFLNB	0,569	2,47	SLITRK4	-0,686	3,1
GREM1	1,123	4,91	SLC11A1	0,722	2,85	RN7SL471P	0,639	1,43	PDE10A	0,565	2,06	CA12	-0,681	2,45
ABCG1	1,061	11,4	MMP19	0,715	2,46	MIR3609	0,637	3,25	AL358075.4	0,565	2,4	GALNT13	-0,663	2,64
STC1	1,05	4,64	RHOU	0,712	2,98	NLRP6	0,633	1,53	SNORA17B	0,563	1,61	CTSG	-0,658	2,74
IL1R2	1,024	4,86	IVNS1ABP	0,705	2,51	TRPM6	0,633	2,41	ERRFI1	0,562	1,6	RNU1-4	-0,651	2,61
B3GNT5	1,01	5,69	CCDC162P	0,704	2,02	SNORA73B	0,632	4,76	SNORA17B	0,562	1,6	LRP1B	-0,632	2,4
AC067817.1	0,991	4,55	TNNT3	0,702	2,7	SNORD89	0,629	2,19	KALRN	0,557	4,38	RAB27B	-0,625	1,43
ADAMTS18	0,939	6,25	RPS16P5	0,701	1,94	SNORA63	0,628	2,4	SNORA73A	0,551	3,07	ADCY2	-0,619	1,86
MIR5690	0,938	3,1	PDE4B	0,696	2,4	NAMPTP1	0,625	1,63	PCLO	0,55	1,94	RNU1-2	-0,616	2,72
COL6A6	0,924	4,26	ST6GALNAC3	0,696	4,24	CEBPD	0,625	1,92	ADGRG1	0,549	2,05	LINC01139	-0,609	1,43
AC007278.1	0,886	2,85	ALG1L13P	0,695	1,53	KDM6B	0,623	1,85	U2AF1	0,548	2,23	CPA3	-0,594	2,75
FOLH1	0,883	2,69	SNORA7B	0,695	2,33	SLC19A2	0,618	2,45	SCARNA5	0,546	2,43	FGFR2	-0,593	1,8
FOSL1	0,851	2,49	LINC-PINT	0,695	3,44	FOSL2	0,614	1,71	MAT2A	0,543	1,33	RNASE6	-0,562	1,75
RNF138P1	0,846	3,43	GRASP	0,693	2,04	SLCO4A1	0,612	1,62	RRP12	0,543	1,43	TPSB2	-0,561	2,37
SVEP1	0,842	6,33	SAA4	0,687	1,6	VEGFC	0,611	1,35	SHANK3	0,542	2,33	P2RY14	-0,542	1,34
RF00019	0,825	2,61	SNORA48	0,685	2,86	NEDD9	0,608	2,03	ABL2	0,541	1,5	B3GNT7	-0,537	1,54
ADGRG3	0,819	2,45	RN7SL1	0,685	5,93	IL18R1	0,604	2,4	NEURL1B	0,54	3,35	TPSAB1	-0,534	2,61
FCAR	0,812	2,46	AL139099.4	0,685	5,93	SLED1	0,602	1,55	DOC2B	0,536	2,06	CCL13	-0,532	1,82
CPZ	0,807	2,23	RAB3C	0,683	2,56	AL356488.2	0,602	6,33	ADM	0,533	1,92	S100A4	-0,53	1,76
C11orf91	0,799	2,17	CEMIP2	0,68	2,7	SCARNA2	0,602	6,33	PELI1	0,531	2,61	ANKS1B	-0,503	1,61
GPR78	0,795	2,16	AC016831.1	0,68	3,7	OSBPL10	0,6	3,25	TNFAIP3	0,53	1,63	ADIRF	-0,503	2,73
OSMR	0,794	3,31	MT1M	0,679	1,83	TRIB1	0,598	1,85	EFNB2	0,529	1,59	LINC01140	-0,501	1,35
RF02271	0,791	2,41	ICAM1	0,678	1,97	IFI44L	0,596	3,17	MCF2L	0,528	2,17			
AL161773.1	0,79	2,54	AP001020.2	0,678	2,02	CLEC4E	0,595	1,33	ARHGAP26	0,524	2,23			
AL356273.3	0,788	2,37	NLRP3	0,678	2,23	RN7SL5P	0,594	3,25	CR1	0,522	1,63			
CYP4F3	0,788	2,53	GABRA2	0,677	1,9	SYN2	0,593	1,85	ALPL	0,521	1,41			
AL596325.2	0,779	2,61	DKK1	0,676	1,45	EXOC3L1	0,592	1,4	SEMA6A	0,516	1,36			
AL138689.1	0,778	2,17	AL022722.1	0,673	2,06	PTX3	0,592	1,59	RALGAPA2	0,515	2,77			
PADI4	0,777	2,28	FAM133CP	0,671	1,55	HSPB6	0,589	2,34	RAPGEF4	0,513	1,8			
AC015819.2	0,776	2,02	EREG	0,67	1,44	AC002558.3	0,588	1,5	MYLIP	0,513	2,42			
SNORD10	0,774	4,86	MIR17HG	0,67	1,53	AVPR1A	0,587	2,17	RASIP1	0,513	2,47			
GRIA1	0,755	2,17	BAALC	0,665	3,29	THBD	0,586	1,41	TRPC6	0,512	1,96			
CCBE1	0,754	3,06	RGS17P1	0,658	1,59	LUCAT1	0,586	1,53	RGPD1	0,511	1,37			
KLF7-IT1	0,754	3,3	THBS4	0,657	3,3	REC8	0,585	1,46	ADAMTSL4-AS1	0,511	1,48			
CA8	0,752	2,94	SEMA6B	0,654	2,5	RPGR	0,584	1,8	ENPEP	0,509	2,41			
LINC01348	0,75	1,85	LRG1	0,649	1,33	ZNF385D	0,584	2,47	PER1	0,509	2,51			
AC122718.2	0,748	2,46	NGFR	0,649	1,5	NABP1	0,582	1,42	CSF3R	0,508	1,62			
SAMSN1	0,747	2,65	APOBEC3A	0,648	1,7	RAPGEF5	0,581	2,53	FADS2	0,504	1,54			
HELZ2	0,746	2,75	PABPC1P3	0,647	1,48	PLSCR1	0,58	2,17	TF	0,503	1,35			
F2RL3	0,742	1,84	NOCT	0,647	1,53	IL1RL1	0,579	1,92	SLFN12L	0,501	1,62			
AL133330.1	0,742	2,39	SLC10A6	0,645	1,48	PIK3R3	0,579	2,47	SLC6A15	-1,221	6,13			
PDGFD	0,742	3,35	SLC26A7	0,643	2,32	FAM107A	0,577	1,36	FCGBP	-1,09	6,25			
AL034397.3	0,741	4,55	AC016831.7	0,643	3,64	MIR6501	0,577	1,72	GABRB2	-0,89	3,64			
AL356356.1	0,739	1,9	UGCG	0,642	1,98	AP000897.1	0,574	1,45	FGFBP2	-0,878	4,83			
IL18RAP	0,739	2,1	AC016831.5	0,642	2,19	TPRIP	0,571	1,62	HPGDS	-0,849	4,02			
AC007278.2	0,736	1,76	AC087286.4	0,641	1,63	C5AR2	0,569	1,55	IGSF21	-0,824	2,46			

Supplementary Data 1: Human LD RNAseq gene expression

Data showing the genes that significantly differ between the control arm and the LD arm based on the fold change and test statistics performed on the RNA-Seq data between conditions (normal arm vs lymphedema arm). *n*=4 women with LD (tissue biopsies from normal arm and LD arm from the same patient. Wald test was used to generate p-values and log2 fold changes. Source data are provided as a Source data file.

Lipids pg/mg of tissue		Precursors	arachidonic acid								
HUMAN ARM Skin		Enzymatic pathways	COX	COX	COX	15-LOX/5-LOX	15-LOX/5-LOX	5-LOX	5-LOX	12-LOX	15-LOX
	PATIENTS	**m (mg)**	**6-K-PGF1A**	**TXB2**	**PGE2**	**LXB4**	**LXA4**	**LTB4**	**5-HETE**	**12-HETE**	**15-HETE**
Ctrl	**1**	**167**	3 208,2	236,9	19 546,6	199,9	8,4	112,5	5 705,0	293 522,6	40 248,7
LD	**1**	**152**	513,5	347,0	3 679,1	119,9	19,8	54,7	3 637,7	133 742,0	8 695,9
Ctrl	**2**	**105**	2 266,8	254,4	9 725,8	64,6	18,0	145,5	2 409,6	307 305,6	44 466,2
LD	**2**	**182**	1 391,5	76,8	11 408,8	21,8	1,8	19,2	912,0	87 542,5	7 173,4
Ctrl	**3**	**157**	1 394,1	105,2	20 312,3	123,6	2,0	67,4	5 309,0	142 912,0	8 548,7
LD	**3**	**150**	131,3	65,7	5 701,0	124,7	2,8	53,3	1 204,3	19 157,5	46 649,4
Ctrl	**4**	**255**	1 858,7	143,7	4 884,2	78,9	9,6	1,9	503,1	31 600,5	15 075,4
LD	**4**	**221**	22,4	34,3	44,0	48,3	3,2	1,2	211,1	3 777,8	1 406,3
Ctrl	**5**	**138**	1 674,3	238,0	6 155,7	139,7	6,9	12,7	718,0	36 564,2	4 198,2
LD	**5**	**182**	235,1	141,8	503,6	72,4	2,3	3,2	167,4	3 143,7	243,0
Ctrl	**6**	**110**	4 674,0	461,1	41 823,5	157,5	14,3	82,2	6 636,5	673 150,9	51 210,4
LD	**6**	**147**	589,3	145,0	16 474,5	65,1	4,8	8,6	1 409,5	75 493,5	4 509,5
LD	**7**	**182**	55,7	27,5	1 682,7	54,3	5,3	6,5	145,2	33 189,7	907,0
LD	**8**	**161**	3 436,2	78,0	3 083,9	39,0	2,7	7,3	347,6	115 554,9	5 114,8
LD	**9**	**174**	238,8	44,4	2 133,9	77,4	2,1	3,3	260,6	6 682,2	2 100,5
LD	**10**	**167**	0,0	1,9	16,2	96,5	3,3	1,6	453,4	12 560,6	9 033,1

Supplementary Table 1: Dosage of AA-derived lipids in human lymphedema skin tissue biopsies. Source data are provided as a Source data file.

Lipids pg/mg of tissue		Precursors	Docosahexaenoic aicd											
HUMAN ARM Skin		Enzymatic pathways	15-LOX/5-LOX	15-LOX/5-LOX	15-LOX/5-LOX	15-LOX/5-LOX	15-LOX/5-LOX	12-LOX	12-LOX/5-LOX	12-LOX	15-LOX/5-LOX	15-LOX	15-LOX	12-LOX
	PATIENTS	m (mg)	RVD1	RVD2	RVD3	RVD4	RVD5	MARESIN	S)-MARESI	MARESIN2	PDX	PD1	17-HDOHE	14-HDOHE
Ctrl	1	167	2,8	2,1	0,0	0,0	15,4	48,9	44,1	16,3	105,4	10,9	23 051,1	20 390,1
LD	1	152	2,5	5,0	1,4	0,0	0,3	26,7	32,6	2,4	111,4	13,8	5 140,4	8 445,5
Ctrl	2	105	1,7	0,7	0,1	0,0	54,3	2,4	47,1	13,9	54,9	9,0	14 009,0	36 910,2
LD	2	182	0,7	4,1	0,0	0,0	7,9	1,4	1,3	4,3	14,4	1,2	1 327,8	4 511,3
Ctrl	3	157	1,6	3,8	0,0	0,0	0,0	1,3	8,4	2,9	19,2	3,4	2 299,7	9 115,2
LD	3	150	1,8	6,7	0,0	0,0	62,6	2,1	8,9	0,1	86,2	1,7	6 879,2	2 892,5
Ctrl	4	255	2,5	2,1	0,0	0,0	39,1	1,3	5,2	0,5	67,2	1,5	3 051,2	3 983,1
LD	4	221	0,5	4,4	0,0	0,0	3,0	0,3	14,6	0,4	9,8	0,0	409,8	454,1
Ctrl	5	138	1,6	2,1	0,0	0,0	3,8	4,8	7,2	5,4	11,2	2,2	1 344,9	6 084,0
LD	5	182	0,4	4,5	0,0	0,0	0,0	1,3	5,1	0,0	4,0	0,0	194,4	698,7
Ctrl	6	110	2,7	4,5	0,0	0,0	24,9	7,0	43,9	11,1	71,8	8,7	9 787,5	57 951,5
LD	6	147	0,2	6,7	0,0	0,0	1,1	5,0	1,4	1,4	10,0	0,5	842,5	5 022,9
LD	7	182	0,0	5,2	0,0	0,0	0,4	0,9	0,6	1,6	3,1	0,5	184,0	1 503,1
LD	8	161	0,2	3,5	0,0	0,0	0,1	1,2	5,8	5,1	4,5	3,9	1 733,7	10 697,7
LD	9	174	0,3	12,8	0,0	0,0	2,1	2,2	0,0	1,1	4,9	0,2	352,9	497,3
LD	10	167	1,3	10,1	0,0	0,0	36,3	1,7	4,6	0,2	44,9	1,3	2 501,0	1 638,9

Supplementary Table 2: Dosage of DHA-derived lipids in human lymphedema skin tissue biopsies. Source data are provided as a Source data file.

Lipids pg/mg of tissue		Precursors	sapentaenoic acid		
HUMAN ARM Skin		Enzymatic pathways	COX/5-LOX; CYP450/5-LOX	COX/5-LOX; CYP450/ 5-LOX	COX; CYP450
	PATIENTS	**m (mg)**	**RVE1**	**RVE2**	**18-HEPE**
Ctrl	**1**	**167**	0,0	5,1	1 802,2
LD	**1**	**152**	0,0	3,9	2 319,5
Ctrl	**2**	**105**	0,0	6,8	1 062,5
LD	**2**	**182**	0,0	2,3	444,1
Ctrl	**3**	**157**	0,0	7,9	808,5
LD	**3**	**150**	0,0	0,7	216,1
Ctrl	**4**	**255**	0,0	2,5	533,8
LD	**4**	**221**	0,0	0,0	160,5
Ctrl	**5**	**138**	0,0	2,3	481,0
LD	**5**	**182**	0,0	0,1	169,0
Ctrl	**6**	**110**	0,0	25,2	2 275,5
LD	**6**	**147**	0,0	0,9	345,2
LD	**7**	**182**	0,0	0,1	110,5
LD	**8**	**161**	0,0	2,9	397,8
LD	**9**	**174**	0,0	0,3	111,4
LD	**10**	**167**	0,0	0,0	329,3

Supplementary Table 3: Dosage of EPA-derived lipids in human lymphedema skin tissue biopsies. Source data are provided as a Source data file.

Lipids pg/mg of tissue		Precursors	arachidonic acid								
HUMAN ARM AT		Enzymatic pathways	COX	COX	COX	15-LOX/5-LOX	15-LOX/5-LOX	5-LOX	5-LOX	12-LOX	15-LOX
	PATIENTS	**m (mg)**	**6-K-PGF1A**	**TXB2**	**PGE2**	**LXB4**	**LXA4**	**LTB4**	**5-HETE**	**12-HETE**	**15-HETE**
Ctrl	**1**	**263**	29,8	165,6	104,9	1 388,6	76,4	555,9	23 489,0	62 598,3	40 156,2
LD	**1**	**301**	30,4	153,5	34,3	75,0	3,8	238,1	4 988,7	27 990,9	4 552,6
Ctrl	**2**	**264**	51,9	112,9	48,4	77,0	3,7	73,1	1 145,5	27 088,0	1 769,8
LD	**2**	**180**	23,4	12,7	4,5	91,1	3,3	9,6	665,0	3 346,6	764,0
Ctrl	**3**	**203**	26,7	47,1	16,7	103,9	4,4	26,5	737,1	10 310,4	956,4
LD	**3**	**198**	8,1	7,2	9,6	88,5	3,6	27,8	528,6	2 323,8	593,0
Ctrl	**4**	**221**	90,9	131,2	122,4	60,9	1,8	13,6	480,9	7 787,9	866,3
LD	**4**	**242**	101,7	253,9	120,0	63,1	2,4	31,8	837,3	30 913,0	1 928,7
Ctrl	**5**	**228**	104,3	141,1	234,2	307,8	12,8	165,9	13 109,1	38 576,8	12 698,2
LD	**5**	**225**	68,1	298,9	105,2	127,9	3,7	121,9	5 178,4	34 549,7	4 803,3
Ctrl	**6**	**244**	31,8	216,9	39,6	82,9	3,9	8,9	796,2	8 499,7	1 355,2
LD	**7**	**167**	84,4	99,4	48,5	72,0	4,1	29,5	622,6	17 077,2	855,1
LD	**8**	**157**	26,7	44,3	24,9	107,3	5,7	9,9	627,6	15 226,2	832,6
LD	**9**	**233**	21,5	176,8	37,2	53,0	1,1	4,6	257,1	7 719,1	409,0
LD	**10**	**249**	3,5	0,4	1,9	94,4	5,7	1,9	518,4	7 389,5	996,4

Supplementary Table 4: Dosage of AA-derived lipids in human lymphedema adipose tissue biopsies. Source data are provided as a Source data file.

Lipids pg/mg of tissue		Precursors	Docosahexaenoic aicd											
HUMAN ARM AT		Enzymatic pathways	15-LOX/5-LOX	15-LOX/5-LOX	15-LOX/5-LOX	15-LOX/5-LOX	15-LOX/5-LOX	12-LOX	12-LOX/5-LOX	12-LOX	15-LOX/5-LOX	15-LOX	15-LOX	12-LOX
	PATIENTS	**m (mg)**	**RVD1**	**RVD2**	**RVD3**	**RVD4**	**RVD5**	**MARESIN1**	**(S)-MARESIN**	**MARESIN2**	**PDX**	**PD1**	**17-HDOHE**	**14-HDOHE**
Ctrl	1	**263**	36,8	27,9	6,1	0,0	7,1	200,5	137,8	13,5	1 248,0	111,3	51 087,9	30 593,7
LD	1	**301**	0,4	12,2	0,1	0,0	0,3	5,7	11,8	1,9	117,8	8,9	6 100,2	8 844,8
Ctrl	2	**264**	0,8	2,2	0,0	0,0	0,0	0,9	14,8	0,6	26,3	4,5	970,5	4 801,9
LD	2	**180**	0,8	4,8	0,0	0,0	0,0	2,5	0,0	0,6	6,3	0,0	337,8	746,6
Ctrl	3	**203**	1,5	3,6	0,0	0,0	0,0	0,5	13,9	0,4	11,7	1,0	844,3	2 036,6
LD	3	**198**	0,0	8,4	0,0	0,0	1,0	1,8	0,0	0,6	10,6	1,0	415,3	566,5
Ctrl	4	**221**	0,0	1,6	0,0	0,0	0,0	0,5	2,9	0,3	3,8	1,0	267,9	1 028,2
LD	4	**242**	0,2	4,2	0,0	0,0	0,0	0,1	13,8	0,0	17,1	0,4	1 016,4	6 075,4
Ctrl	5	**228**	0,2	30,0	0,4	0,0	1,6	5,3	18,2	0,3	183,3	12,4	11 581,7	9 881,9
LD	5	**225**	4,1	27,1	0,0	0,0	0,0	3,2	20,3	1,2	102,4	6,3	4 349,2	8 495,1
Ctrl	6	**244**	0,7	6,5	0,0	0,0	0,0	1,2	4,1	0,7	15,8	2,5	907,8	1 634,1
LD	7	**167**	0,5	4,3	0,0	0,0	0,0	1,9	9,1	0,0	8,9	0,3	642,0	4 786,1
LD	8	**157**	0,6	10,8	0,0	0,0	0,4	0,0	5,0	0,6	5,8	0,3	618,6	3 932,6
LD	9	**233**	0,2	2,6	0,0	0,0	0,1	0,0	0,3	0,0	0,5	0,1	111,8	793,3
LD	10	**249**	1,2	8,5	0,0	0,0	0,0	5,3	3,5	1,4	10,2	2,2	624,2	1 244,1

Supplementary Table 5: Dosage of DHA-derived lipids in human lymphedema adipose tissue biopsies. Source data are provided as a Source data file.

Lipids pg/mg of tissue		Precursors	Eicosapentaenoic acid		
HUMAN ARM AT		Enzymatic pathways	COX/5-LOX; CYP450/5-LOX	COX/5-LOX; CYP450/5-LOX	COX; CYP450
	PATIENTS	**m (mg)**	**RVE1**	**RVE2**	**18-HEPE**
Ctrl	**1**	**263**	0,0	16,4	26 007,9
LD	**1**	**301**	0,0	0,9	2 771,4
Ctrl	**2**	**264**	0,0	0,5	944,9
LD	**2**	**180**	0,0	0,0	492,0
Ctrl	**3**	**203**	0,0	0,2	315,3
LD	**3**	**198**	0,0	0,0	222,3
Ctrl	**4**	**221**	0,0	0,0	376,1
LD	**4**	**242**	0,0	0,1	821,3
Ctrl	**5**	**228**	0,0	3,2	7 481,8
LD	**5**	**225**	0,0	0,0	2 181,2
Ctrl	**6**	**244**	0,0	0,0	543,6
LD	**7**	**167**	0,0	0,0	318,9
LD	**8**	**157**	0,0	0,0	250,0
LD	**9**	**233**	0,0	0,0	146,7
LD	**10**	**249**	0,0	0,1	510,2

Supplementary Table 6: Dosage of EPA-derived lipids in human lymphedema adipose tissue biopsies. Source data are provided as a Source data file.

Lipids: pg/mg tissue			arachidonic acid								
**Mice limb AT**			COX	COX	COX	15-LOX/5-LOX	15-LOX/5-LOX	5-LOX	5-LOX	12-LOX	15-LOX
	Mouse#		**6-K-PGF1A**	**TXB2**	**PGE2**	**LXB4**	**LXA4**	**LTB4**	**5-HETE**	**12-HETE**	**15-HETE**
2 weeks	1	LD limb	2,77	2,24	6,83	0,24	0,02	0,13	13,43	242,20	81,78
	2		2,46	1,15	2,92	0,09	0,09	0,18	11,78	159,20	58,04
	3		2,92	2,29	4,34	0,04	0,16	0,21	23,86	210,11	87,02
	4		2,15	1,69	4,83	0,47	0,06	0,15	19,44	411,21	137,44
	5		6,19	4,50	9,05	0,79	0,19	0,29	43,09	425,15	181,99
	1	Ctrl limb	4,35	1,09	8,71	0,29	0,16	0,04	19,71	270,62	71,50
	2		10,13	1,94	10,20	0,00	0,09	0,13	28,67	387,02	138,90
	3		6,50	2,05	9,01	0,68	0,06	0,18	19,63	413,87	135,16
	4		1,13	0,77	3,88	0,09	0,04	0,11	55,59	473,21	202,21
	5		11,03	2,69	14,00	0,35	0,08	0,05	32,16	304,53	115,82
	1	No surgery	2,95	4,77	9,18	0,00	0,16	0,53	57,54	506,10	211,11
	2		0,01	0,54	1,22	0,00	0,13	0,19	28,79	202,85	88,05
	3		0,76	0,90	2,24	0,08	0,02	0,12	18,25	360,14	109,78
	4		0,00	0,43	1,12	0,01	0,07	0,10	16,52	248,80	76,83
	5		0,80	0,77	2,30	0,38	0,07	0,04	14,93	152,17	60,49
8 weeks	1	LD limb	2,52	2,35	5,86	0,34	0,12	5,78	48,75	464,65	146,09
	2		1,30	1,57	3,35	0,12	0,05	3,23	21,39	91,39	37,29
	3		2,02	0,97	4,83	0,27	0,03	0,81	12,79	33,19	17,21
	4		1,48	1,20	2,97	0,16	0,07	0,80	13,08	64,09	25,70
	5		8,63	2,23	27,08	0,14	0,01	0,11	5,66	136,99	43,74
	1	Ctrl limb	11,35	5,57	27,03	0,45	0,18	0,51	16,03	373,16	78,99
	2		2,42	0,61	6,67	0,37	0,05	0,26	8,05	54,65	15,01
	3		0,47	0,25	4,38	0,18	0,01	0,10	4,23	10,04	3,93
	4		3,44	1,51	13,12	0,04	0,02	0,13	6,69	99,57	23,40
	5		11,28	2,21	17,56	0,12	0,02	0,22	6,17	54,65	19,69
	1	No surgery	5,69	3,35	7,55	0,35	0,03	1,80	23,37	80,59	39,16
	2		3,97	2,01	10,44	0,41	0,03	1,13	13,46	164,18	56,02
	3		1,39	0,58	4,30	0,30	0,01	0,01	2,47	17,39	6,68
	4		5,87	3,96	17,81	0,16	0,06	2,58	40,52	309,69	117,38
	5		3,15	1,88	4,42	0,12	0,06	0,75	17,40	136,64	49,48

Supplementary Table 7: Dosage of AA-derived lipids in mice lymphedema adipose tissue biopsies 2 and 8 weeks after surgery. Source data are provided as a Source data file.

Lipids: pg/mg tissue			Docosahexaenoic aicd											
Mice limb AT			15-LOX/5-LOX	15-LOX/5-LOX	15-LOX/5-LOX	15-LOX/5-LOX	15-LOX/5-LOX	12-LOX	12-LOX/5-LOX	12-LOX	15-LOX/5-LOX	15-LOX	15-LOX	12-LOX
	Mouse#		**RVD1**	**RVD2**	**RVD3**	**RVD4**	**RVD5**	**MARESIN1**	**7(S)-MARESIN1**	**MARESIN2**	**PDX**	**PD1**	**17-HDOHE**	**14-HDOHE**
2 weeks	1	LD limb	0,04	0,00	0,01	0,19	0,31	0,14	0,01	0,96	1,32	0,05	110,54	161,31
	2		0,02	0,00	0,01	0,23	0,47	0,04	0,06	1,55	1,74	0,10	72,36	130,12
	3		0,09	0,00	0,02	0,46	0,57	0,10	0,00	1,98	1,29	0,12	60,12	100,71
	4		0,03	0,00	0,01	0,57	0,36	0,09	0,00	1,77	2,61	0,05	230,34	289,94
	5		0,17	0,00	0,12	0,37	1,16	0,38	0,49	5,31	2,71	0,20	145,95	242,87
	1	Ctrl limb	0,02	0,00	0,03	0,35	0,14	0,11	0,07	0,93	1,17	0,13	105,05	144,40
	2		0,09	0,00	0,00	0,23	0,44	0,00	0,11	1,44	2,36	0,10	284,49	370,92
	3		0,04	0,00	0,00	0,41	0,40	0,02	0,09	1,07	2,84	0,08	301,54	370,99
	4		0,08	0,00	0,06	0,46	0,86	0,11	0,10	2,88	2,86	0,21	229,29	312,67
	5		0,06	0,00	0,00	0,63	0,23	0,02	0,14	1,28	1,54	0,10	136,67	232,51
	1	No surgery	0,10	0,00	0,04	0,89	0,54	0,37	0,32	2,85	2,51	0,15	212,80	281,37
	2		0,07	0,00	0,03	0,42	0,23	0,10	0,42	1,21	1,04	0,10	76,90	112,81
	3		0,03	0,00	0,04	0,32	0,50	0,17	0,23	2,16	2,46	0,13	145,01	231,41
	4		0,02	0,00	0,03	0,29	0,45	0,04	0,09	1,28	1,31	0,06	78,43	120,46
	5		0,12	0,00	0,03	0,49	0,30	0,08	0,04	0,86	0,96	0,02	76,50	124,42
8 weeks	1	LD limb	0,02	0,00	0,01	0,16	0,95	0,06	0,25	1,40	3,13	0,29	322,43	419,27
	2		0,01	0,00	0,00	0,08	0,34	0,00	0,02	0,22	0,73	0,05	76,10	89,71
	3		0,02	0,00	0,00	0,04	0,05	0,02	0,02	0,03	0,13	0,02	14,81	22,20
	4		0,05	0,00	0,01	0,10	0,18	0,00	0,09	0,13	0,64	0,04	42,84	56,78
	5		0,01	0,00	0,00	0,08	0,15	0,01	0,00	0,34	1,23	0,00	62,42	88,77
	1	Ctrl limb	0,05	0,00	0,00	0,32	1,60	0,09	0,12	0,85	2,96	0,11	221,93	309,83
	2		0,02	0,00	0,00	0,04	0,10	0,00	0,02	0,05	0,32	0,03	31,81	48,71
	3		0,02	0,00	0,00	0,02	0,01	0,00	0,00	0,01	0,12	0,03	5,99	8,07
	4		0,01	0,00	0,00	0,07	0,24	0,00	0,05	0,15	0,71	0,07	62,47	76,34
	5		0,01	0,00	0,00	0,03	0,08	0,00	0,02	0,10	0,39	0,02	29,18	36,46
	1	No surgery	0,02	0,00	0,00	0,04	0,51	0,00	0,06	0,20	1,39	0,10	95,69	118,93
	2		0,02	0,00	0,00	0,08	0,50	0,01	0,03	0,26	1,86	0,13	107,97	137,05
	3		0,00	0,00	0,00	0,02	0,09	0,01	0,00	0,03	0,47	0,03	26,45	36,92
	4		0,02	0,00	0,00	0,13	0,76	0,03	0,06	0,84	2,14	0,11	190,02	260,57
	5		0,01	0,00	0,00	0,06	0,47	0,00	0,11	0,22	1,87	0,18	84,49	126,56

Supplementary Table 8: Dosage of DHA-derived lipids in mice lymphedema adipose tissue biopsies 2 and 8 weeks after surgery. Source data are provided as a Source data file.

Lipids: pg/mg tissue			Eicosapentaenoic acid		
**Mice limb AT**			COX/5-LOX; CYP450/5-LOX	COX/5-LOX; CYP450/5-LOX	COX; CYP450
	Mouse#		**RVE1**	**RVE2**	**18-HEPE**
2 weeks	1	LD limb	0,06	0,006	0,74
	2		0,03	0,019	0,31
	3		0,28	0,009	0,48
	4		0,11	0,003	0,74
	5		0,09	0,059	0,88
	1	Ctrl limb	0,05	0,000	0,95
	2		0,10	0,000	1,70
	3		0,07	0,000	0,63
	4		0,15	0,028	2,04
	5		0,07	0,000	1,66
	1	No surgery	0,57	0,023	1,63
	2		0,05	0,023	0,37
	3		0,05	0,011	0,40
	4		0,20	0,000	0,26
	5		0,10	0,005	0,90
8 weeks	1	LD limb	0,01	0,011	1,40
	2		0,00	0,012	0,62
	3		0,01	0,005	0,71
	4		0,01	0,005	0,66
	5		0,02	0,008	0,39
	1	Ctrl limb	0,01	0,006	0,77
	2		0,01	0,003	0,43
	3		0,01	0,000	0,30
	4		0,01	0,000	0,39
	5		0,01	0,005	0,17
	1	No surgery	0,00	0,015	0,88
	2		0,00	0,012	0,78
	3		0,02	0,000	0,32
	4		0,01	0,020	2,00
	5		0,01	0,021	1,08

Supplementary Table 9: Dosage of EPA-derived lipids in mice lymphedema adipose tissue biopsies 2 and 8 weeks after surgery. Source data are provided as a Source data file.

Supplementary Figure 8

Blot corresponding to Figure 2e

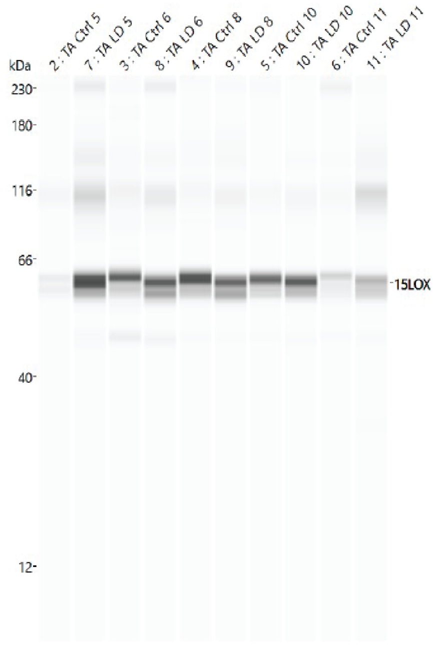

Blot corresponding to Figure 4i

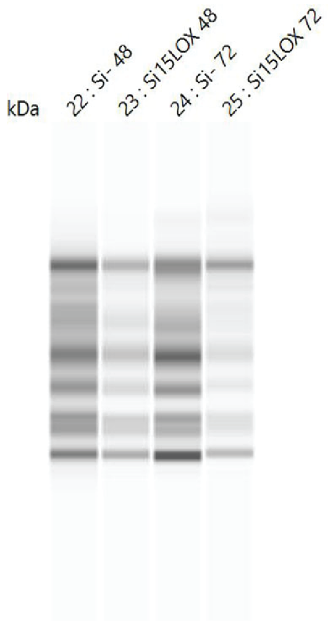

Blot corresponding to Supplementary Figure 3i

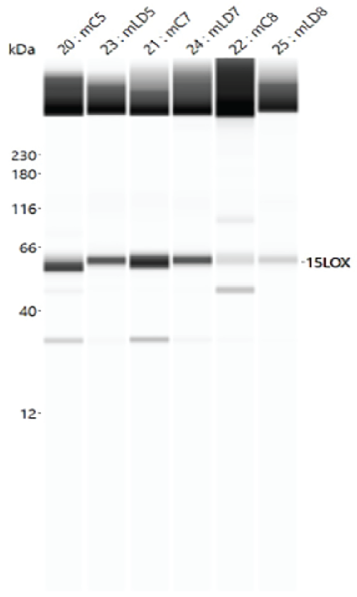

	Forward primers	Reverse primes
15-LO	5’-AGCCCCAACTAATGACACGG-3’	5’- TGACCCCATCCTCACATTGC-3’
CCL21	5’-CCTTGCCACACTCTTTCTCCC-3’	5’-CAAGGAAGAGGTGGGGTGTA-3’
SPHK1	5’-CCGGTAGATGCACACCTTGT-3’	5’-TGGGTGCAGCAAACATCTCA-3’
SPHK2	5’-CTAGATCGCCCTGACTGGGA-3’	5’-CTGGCTCAAATCCCCCGTG-3’
S1PR1	5’-CCCCATGTGAAAGCGTCTCT-3’	5’-TGCACACACTCACTTGGGTT-3’
LTBR	5’-TGGAAGAGCCACCCTTCTCT-3’	5’-AGCAGTGGCTGTACCAAGTC-3’
ICAM1	5’-CTCCAATGTGCCAGGCTTG-3’	5’-CAGTGGGAAAGTGCCATCCT-3’
VCAM1	5’- TTCCCTAGAGATCCAGAAATCGAG-3’	5’-CCTGCAGCTTACAGTGACAGAGC-3’.
IFNa	5’- ACCTCAGGAACAAGAGAGCC-3’	5’-CTTCTCCTGCGGGAATCCAA-3’
IFNb	5’- CCAGCTCCAAGAAAGGACGA-3'	5’-TGGATGGCAAAGGCAGTGTA-3'
LTBR	5’- GGCACCCAGAGGGAGAAGA-3’	5’-CTGCCAGGTCAGGGAAATGT-3’

Supplementary Table 10: List of oligonucleotides
