# Supplementary material for: 15-Lipoxygenase promotes resolution of inflammation in lymphedema by controlling Treg cell function through IFN-β
Source: Nat Commun. 2024 Jan 4;15:221. doi: 10.1038/s41467-023-43554-y (PMC10766617; doi:10.1038/s41467-023-43554-y)
Supplement: Supplementary file 3 — Description of Additional Supplementary Files [file 41467_2023_43554_MOESM3_ESM.pdf]

Description of Additional Supplementary Files

Supplementary Data 1: These data correspond to the genes that significantly differ between the control arm and the LD arm based on the fold change (0.5) and test statistics performed on the RNA-Seq data.
