# Supplementary material for: 15-Lipoxygenase promotes resolution of inflammation in lymphedema by controlling Treg cell function through IFN-β
Source: Nat Commun. 2024 Jan 4;15:221. doi: 10.1038/s41467-023-43554-y (PMC10766617; doi:10.1038/s41467-023-43554-y)
Supplement: Supplementary file 6 — Source Data [file 41467_2023_43554_MOESM6_ESM.zip › Supplementary Informations.docx]

**SUPPLEMENTARY INFORMATION**


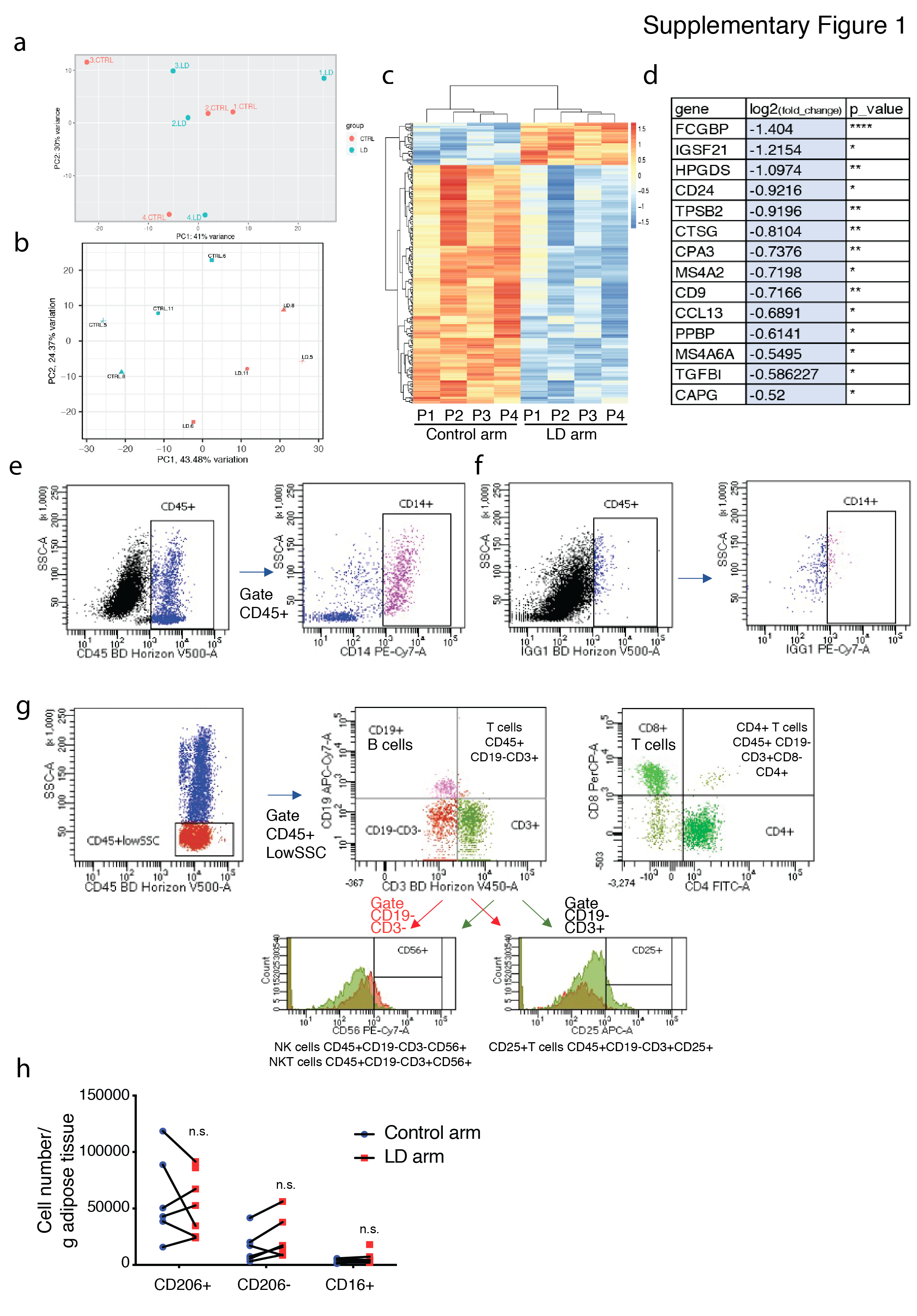


**Supplementary Figure 1: Human LD RNAseq expression profile**

**a**. Principle component analysis (PCA) plot for RNA seq data of biological replicates of biopsies from control arm (CTRL) and lymphedema arm (LD) on each patient. **b**. PCA plot realized after the batch effect correction showing clear separation by condition (CTRL versus LD). **c**, Hierarchical clustering heatmap of the differentially expressed genes between the CTRL and the LD condition of the RNA-seq data, after the batch effect of the individual n.1 was corrected. **d**, Downregulated genes associated with immune response. **e-g**. Representative dot plots of the flow cytometry analyses are shown starting from viable cells gated on side scatter (SSC)/forward scatter (FSC) of the stroma vascular cells (not shown). **e**. Macrophages, defined as CD45+/CD14+ are gated from CD45+ cells from the CD45/SSC dot plot, **f**. positioned from dot plots obtained with respective isotype controls. **g**. T lymphocytes, defined as CD45+/CD19-/CD3+ and B lymphocytes defined as CD45+/CD3-/CD19+ are gated from CD45+/low SCC. T lymphocytes are further divided into CD4+ T lymphocytes and CD8+ T lymphocytes. NK (CD3-/CD56+) and NKT (CD3+/CD56+) are gated from CD19-/CD3- cells and CD19-/CD3+ cells respectively and CD25+ T cells from CD19-/CD3+ cells. **h**. Flow cytometry analysis of CD206+ and CD16+ cells in LD dermolipectomies.

For **a-d**, *n*=4 women with LD (tissue biopsies from normal arm and LD arm from the same patient). For **e-h**, *n*=6 women with LD (tissue biopsies from normal arm and LD arm from the same patient). (**e**) Wald test was used to generate p-values and log2 fold changes. (**h**) *P* values are derived from two-way ANOVA. Source data are provided as a Source data file.


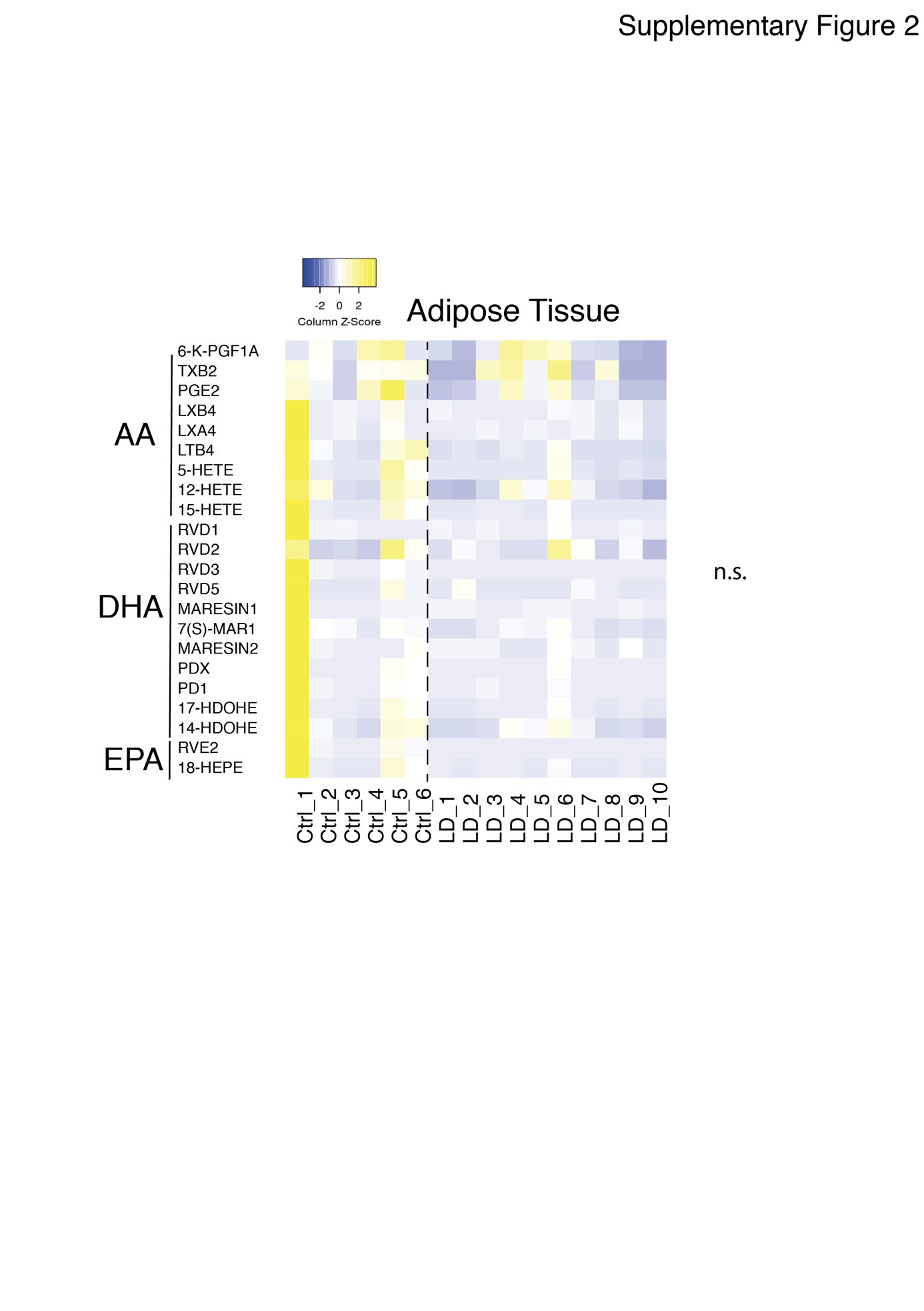


**Supplementary Figure 2: Human LD lipidomic analysis**

Heatmap of lipid mediators derived from arachidonic acid (AA), Docosahexaenoic acid (DHA) and Eicosapentaenoic acid (EPA) analysis in lymphedematous adipose tissue from women who developed LD after breast cancer. *n*=4 women with LD (tissue biopsies from normal arm and LD arm from the same patient) and n=6 women with LD (LD arm tissue biopsies only). Source data are provided as Supplementary tables 3-5.

**
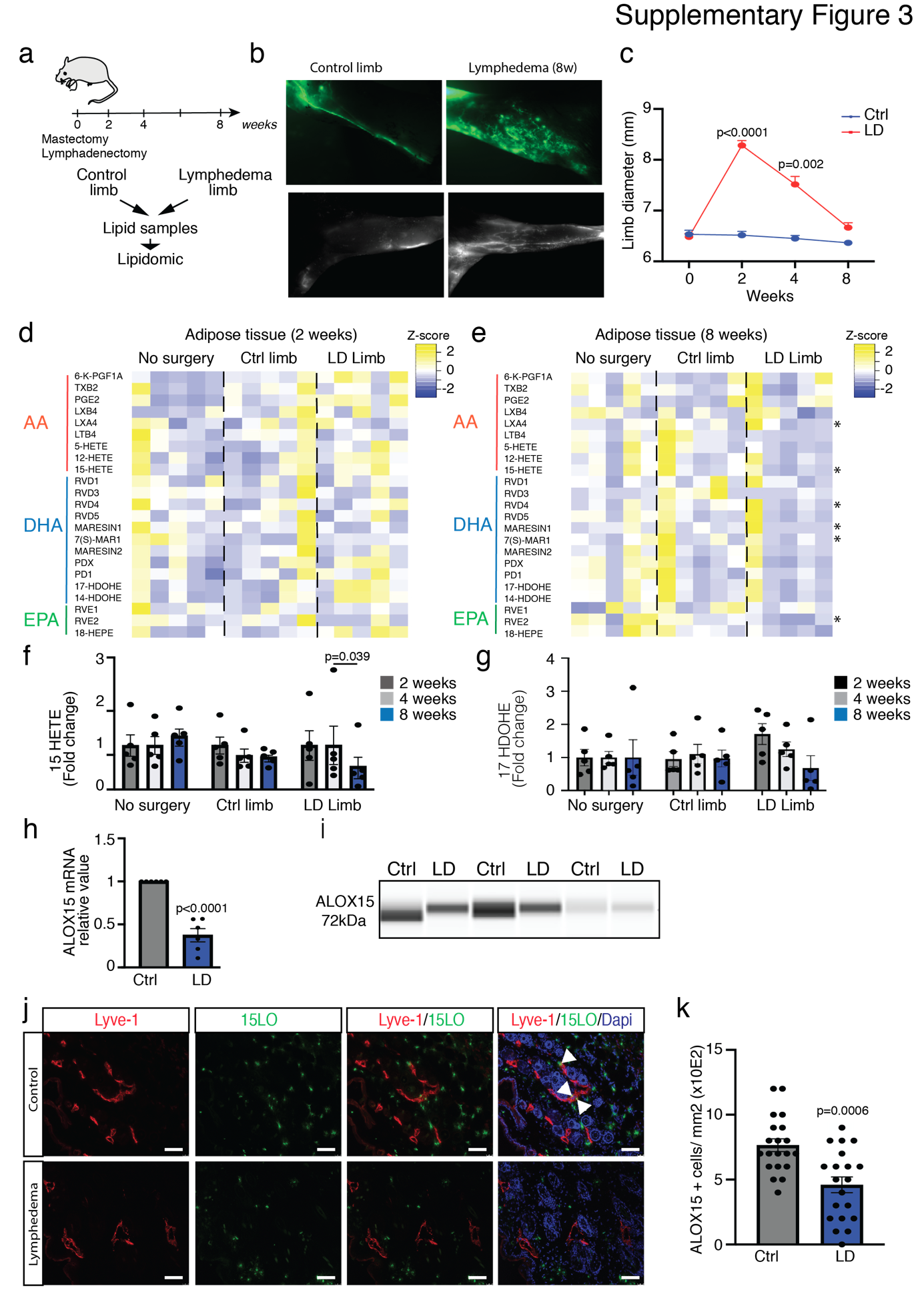
**

**Supplementary Figure 3: Downregulation of ALOX15 in a mouse model of lymphedema**

**a**, Schematic representation of the experimental procedure. **b**, Lymphography of the limb from mice with LD. **c**, Quantification of the limb diameter in mice with LD. d-e, Heatmap of lipid mediators derived from arachidonic acid (AA), Docosahexaenoic acid (DHA) and Eicosapentaenoic acid (EPA) analysis in lymphedematous adipose tissue from mice 2 weeks (**d**) and 8 weeks (**e**) post-surgery. **f**, 15-HETE dosage in mouse LD (*P<0.05). **g**, Quantification of 17-HDOHE in mouse LD. **h**, *Alox15* mRNA expression in mouse LD. **i**, 15-LO protein expression in mouse LD. **j**, Immunodetection of 15-LO (green) and LYVE-1 (red) in lymphedematous skin (Scale bar: 50μm). **k**, Quantification of the 15-LO-positive cells in mouse LD (***P<0.001). For **a-d**, *n*=4 women with LD (tissue biopsies from normal arm and LD arm from the same patient). For **b,c** *n*=6 mice per group. For **d,g** *n*=5 mice per group. For **h** *n*=6-7 mice per group. For **i** *n*=3 mice. For **j,k** *n*=4 mice per group. Data are shown as mean ± s.e.m. (**f,g**) *P* values are derived from two-way ANOVA. (**d,e)** *P* values are derived from unpaired t-test. (**f,g**) *P* values are derived from one-way ANOVA. (**k)** *P* values are derived from unpaired t-test. Source data are provided as a Source data file.

**
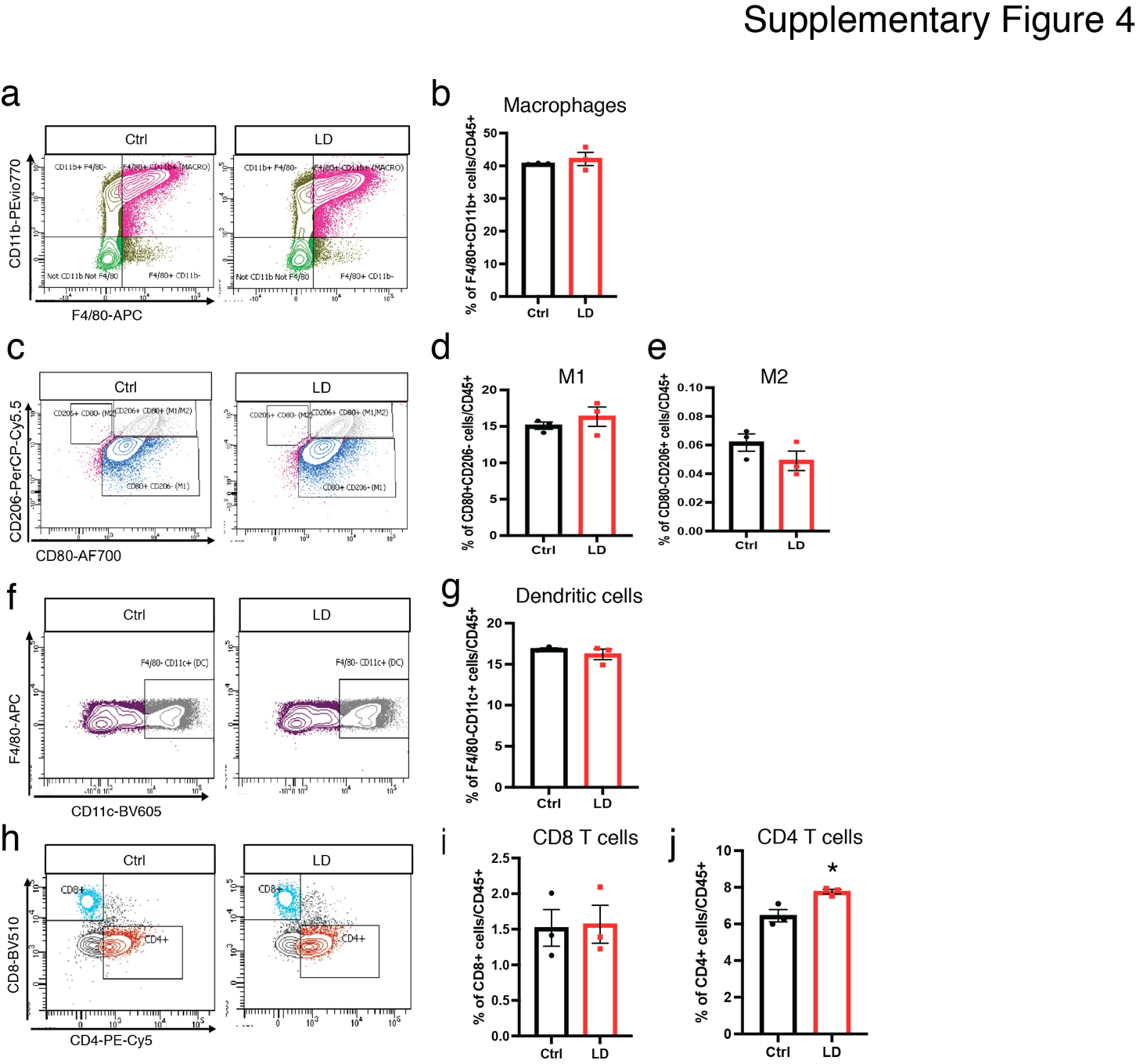
**

**Supplementary Figure 4: Flow cytometry analysis of AT samples in mice LD**

**a**, Representative FACS plots of LD macrophages. Gating method for the identification of macrophages (F4/80+/CD11b+). **b**, Quantification showing the proportion of macrophages in Ctrl and LD AT. **c**, Representative FACS plots of LD M1 and M2 macrophages. Gating method for the identification of macrophages (CD80+/CD206+). **d**, Quantification showing the proportion of M1 and M2 macrophages in Ctrl and LD AT. **e**, Representative FACS plots of LD dendritic cells. Gating method for the identification of dendritic cells (CD11c+/F4/80-). **f**, Representative FACS plots of LD dendritic cells. **g**, Quantification showing the proportion of dendritic cells in Ctrl and LD AT. **h**, Representative FACS plots of LD lymphocytes. Gating method for the identification of TCD4+ and TCD8+ cells. **i**, Quantification showing the proportion of CD8+ T cells in Ctrl and LD AT. **j**, Quantification showing the proportion of CD4+ T cells in Ctrl and LD AT. For **a-j,** *n*=3 mice per group. (**b,d,e,g,I,j**) *P* values are derived from unpaired t-test. Source data are provided as a Source data file.

**
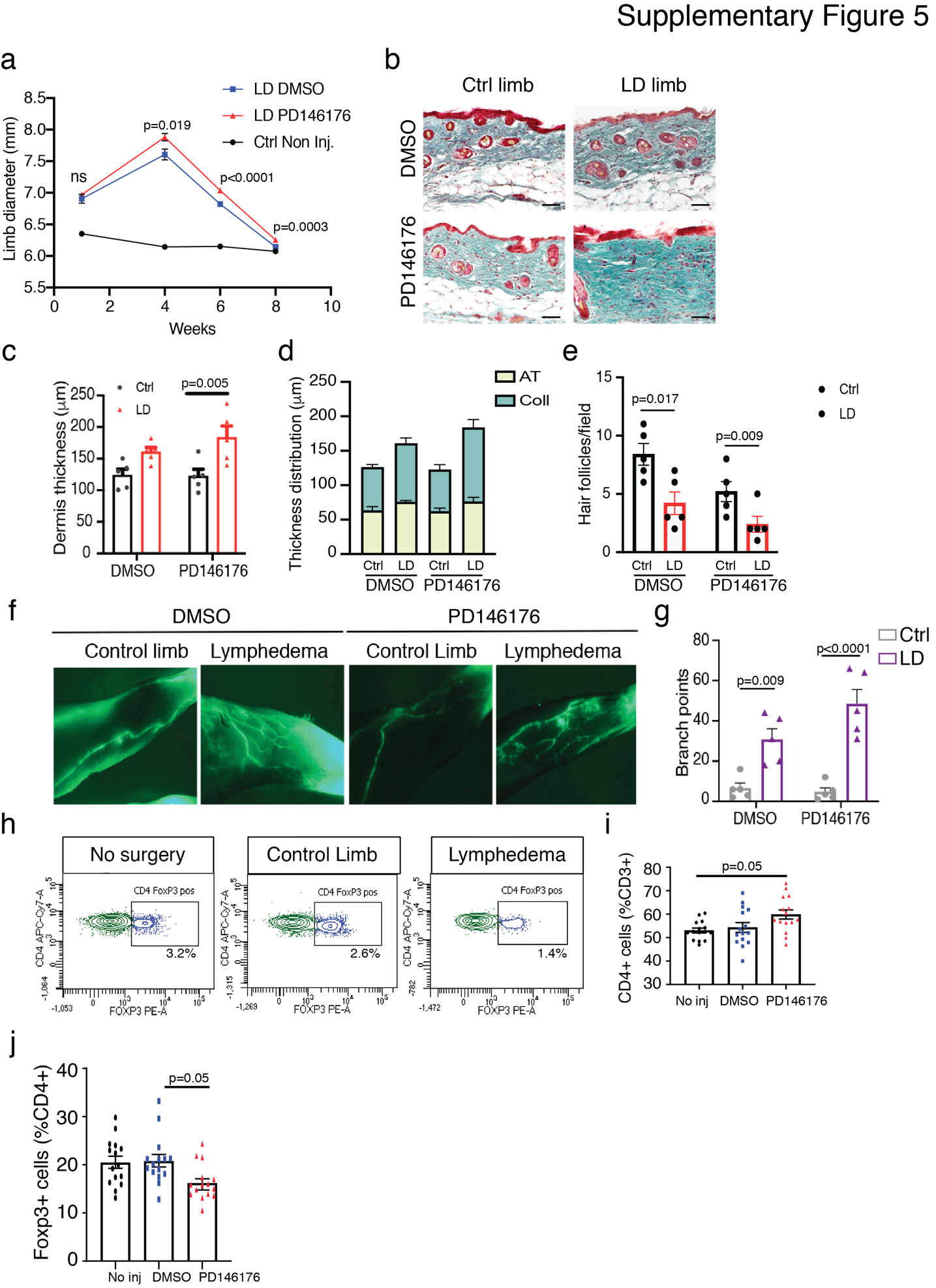
**

**Supplementary Figure 5: ALOX15 inhibitors reduce Treg number in LD**

**a**, Quantification of the limb diameter in mice with LD treated with 15-LO inhibitor (PD146176). **b**, Masson’s trichrome coloration of the lymphedematous skin. **c**, Quantification of dermis thickness in mice with LD treated with PD146176. **d**, Skin thickness repartition related to collagen (Coll) vs adipose tissue (AT). **e**, Hair follicle quantification in LD skin. **f**, Lymphography of the limb from mice with LD treated with PD146176. **g**, Quantification of lymphatic branch point in the limb from mice with LD treated with PD146176. **h**, Representative FACS plots of LD CD4+ lymphocytes and Treg cells in LD. **i-j**, Flow cytometry analysis of CD4+ (**i**) and Foxp3+ (**j**) cell populations in spleen from mice treated with PD146176. For **a**  *n*=10 mice per group. For **b-j** *n*=5 mice per group. Data are shown as mean ± s.e.m. (**a,c,e,g**) *P* values are derived from two-way ANOVA. (**i,j)** *P* values are derived from one-way ANOVA. Source data are provided as a Source data file.

**
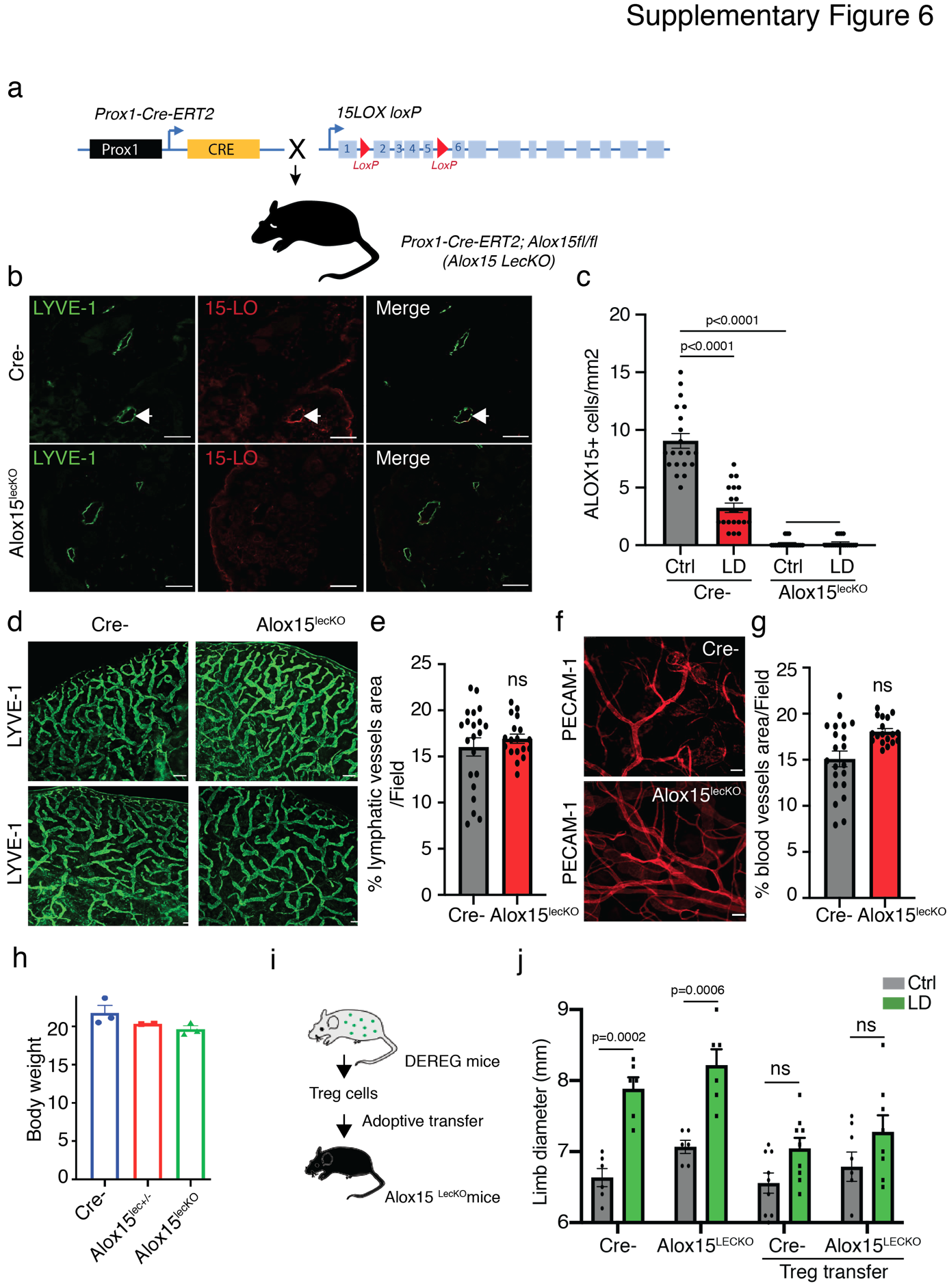
**

**Supplementary Figure 6: ALOX15 lymphatic knock down had no effect on basal vascular networks.**

**a**, Schematic representation of ALOX15^LECKO^ transgenic mice. **b**, Immunodetection of 15-LO (red) and Lyve-1 (green) in ALOX15^LECKO^ and Cre- control littermates’ skin (Scale bar: 50μm). **c**, Quantification of ALOX15-positive LEC in mice’s skin. **d**, Lyve-1 immunodetection of the lymphatic dermal network in ALOX15^LECKO^ mice (Scale bar: 25μm). **e**, Quantification of dermal lymphatic vessel density in ALOX15^LECKO^ mice. **f**, PECAM-1 immunodetection of the blood vessels in ALOX15^LECKO^ mice (Scale bar: 25μm). **g**, Quantification of dermal blood vessel density in ALOX15^LECKO^ mice. **h**, ALOX15 knock-down in the lymphatic system has no effect on body weight. **i**, Schematic representation of Treg cells adoptive transfer from DEREG mice to ALOX15^LECKO^ mice. **j**, Quantification of the limb diameter in ALOX15^LECKO^ mice with LD after Treg transfer in the limb AT. For **b-g**  *n*=10 mice per group. For **h** *n*=3 mice per group. For **j** *n*=6-9 mice per group. Data are shown as mean ± s.e.m. (**c,j**) *P* values are derived from two-way ANOVA. (**h)** *P* values are derived from one-way ANOVA. (**e,g**) *P* values are derived from unpaired t-test. Source data are provided as a Source data file.

**
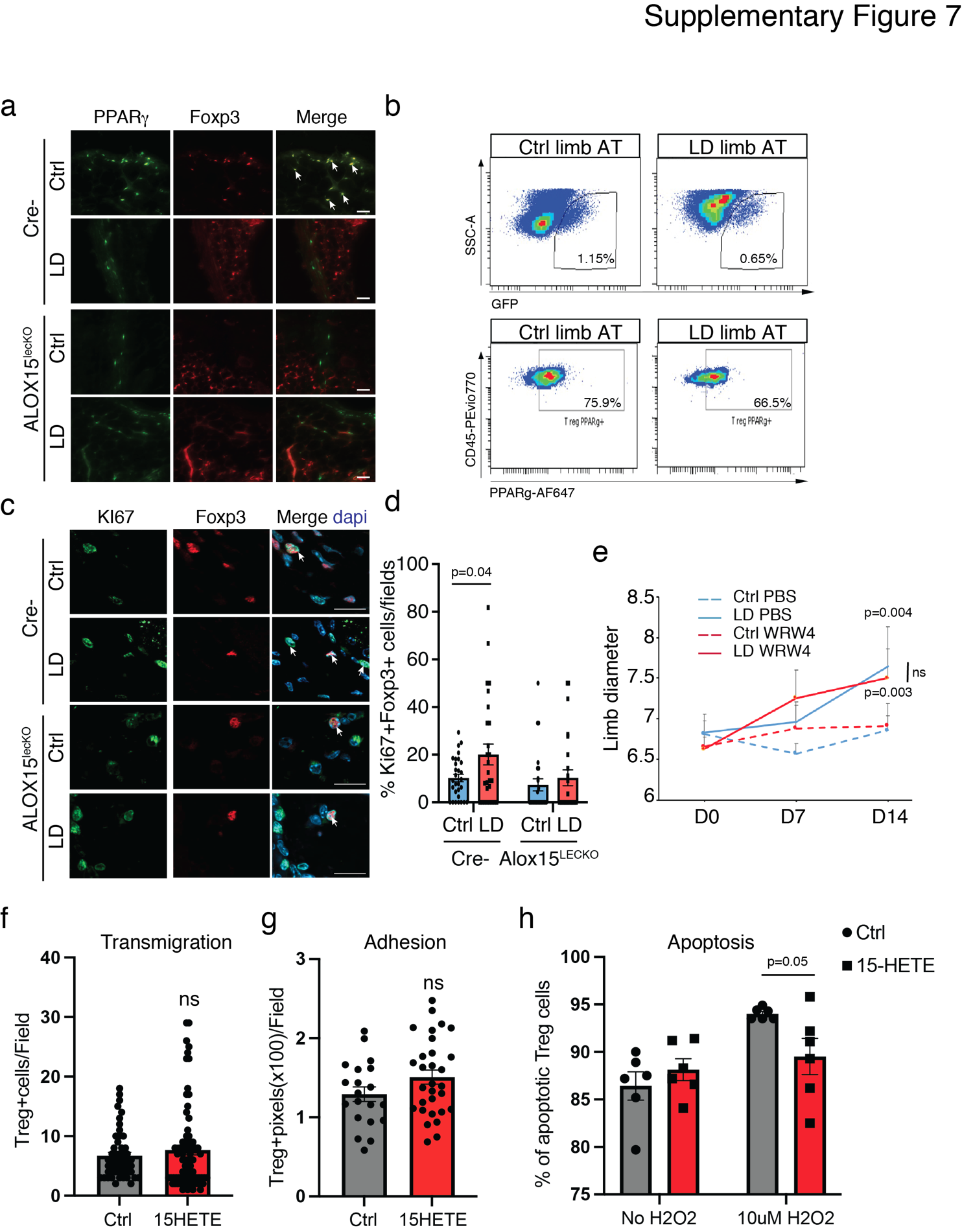
**

**Supplementary Figure 7: Lymphatic endothelium protects Treg cells from apoptosis**

**a**, Immunodetection of PPARg (green) and Foxp3 (red) in ALOX15^LECKO^ and Cre- control littermates’ skin (Scale bar: 50μm). **b**, Representative FACS plots of AT from Ctrl and LD Treg cells (GFP+) and PPARg+ cells. Gating method for the identification of PPARg+ Treg cells. **c**, Immunodetection of proliferative Treg cells using KI67 (green) and Foxp3 (red) in ALOX15^LECKO^ and Cre- control littermates’ skin (Scale bar: 50μm). **d**, Quantification of the percentage of KI67-positive Treg cells. **e**, Quantification of the limb diameter in ALOX15^LECKO^ mice with LD treated with WRW4 inhibitor. **f**, Quantification of Treg trans-lymphatic endothelial migration. **g**, Quantification of Treg cells adhesion to the lymphatic endothelial monolayer. **h**, Quantification of apoptotic Treg cells treated with 15-HETE.

For **a-d**  *n*=5 mice per group. For **e** *n*=7 mice per group. For **f-f** *n*=3 independent replicates. Data are shown as mean ± s.e.m. (**d**) *P* values are derived from multiple Mann-Whitney analysis. (**e)** *P* values are derived from two-way ANOVA. (**f-h**) *P* values are derived from unpaired t-test. Source data are provided as a Source data file.

**Supplementary Data 1: Human LD RNAseq gene expression**

Data showing the genes that significantly differ between the control arm and the LD arm based on the fold change and test statistics performed on the RNA-Seq data between conditions (normal arm vs lymphedema arm). *n*=4 women with LD (tissue biopsies from normal arm and LD arm from the same patient. Wald test was used to generate p-values and log2 fold changes. Source data are provided as a Source data file.


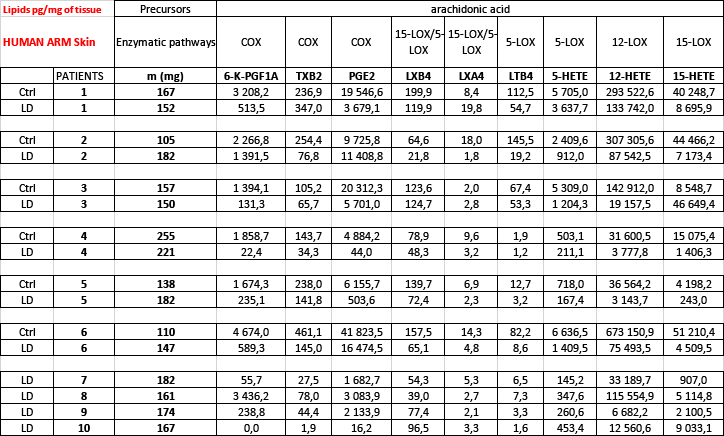


**Supplementary Table 1**: Dosage of AA-derived lipids in human lymphedema skin tissue biopsies. Source data are provided as a Source data file.


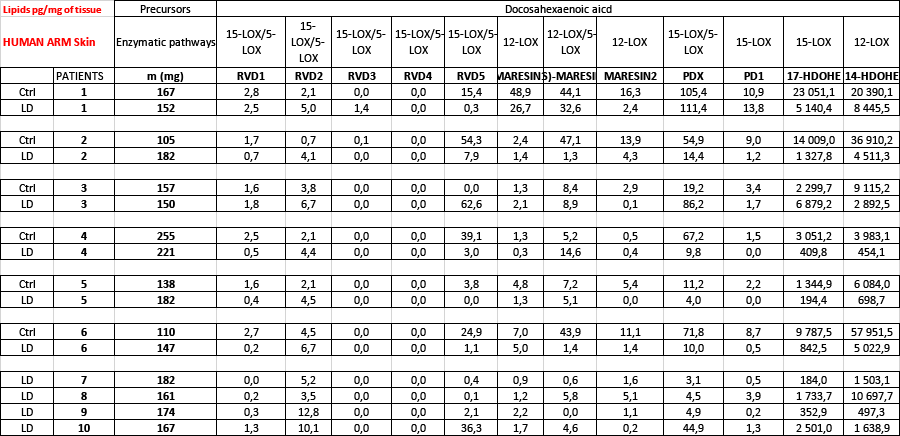


**Supplementary Table 2**: Dosage of DHA-derived lipids in human lymphedema skin tissue biopsies. Source data are provided as a Source data file.


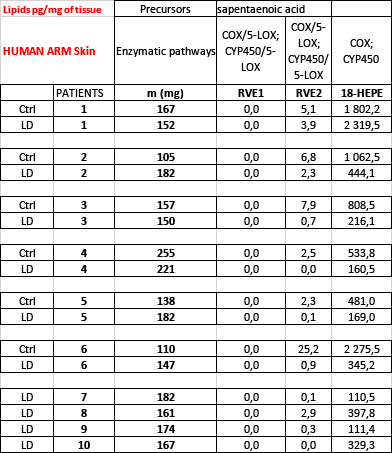


**Supplementary Table 3**: Dosage of EPA-derived lipids in human lymphedema skin tissue biopsies. Source data are provided as a Source data file.


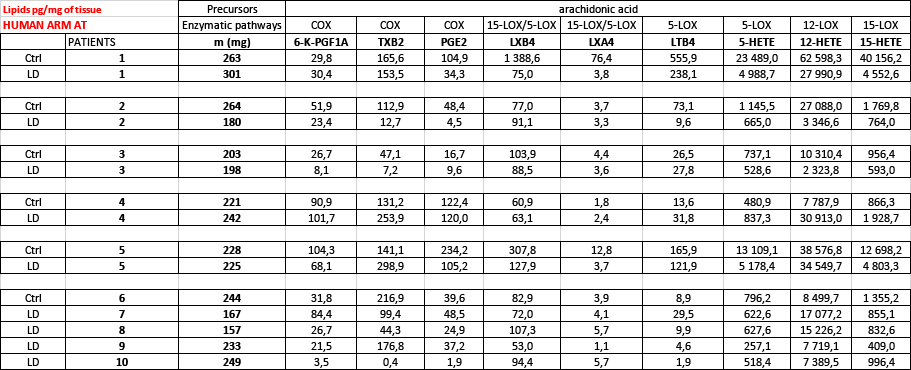


**Supplementary Table 4**: Dosage of AA-derived lipids in human lymphedema adipose tissue biopsies. Source data are provided as a Source data file.


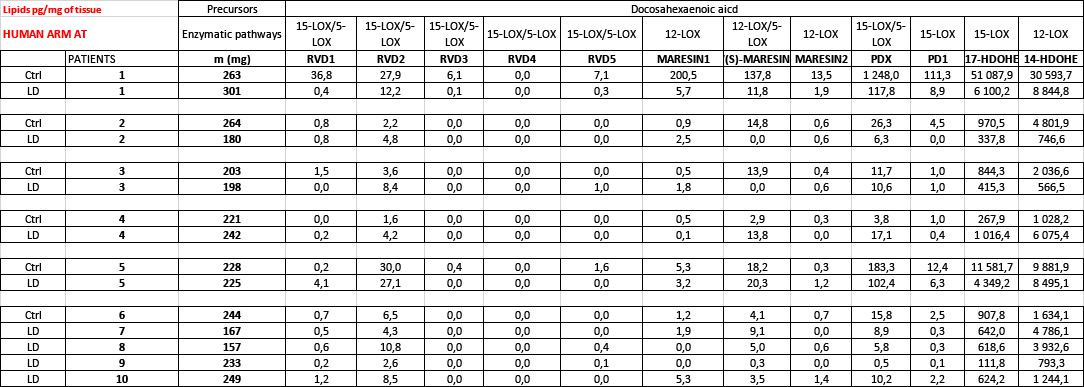


**Supplementary Table 5**: Dosage of DHA-derived lipids in human lymphedema adipose tissue biopsies. Source data are provided as a Source data file.


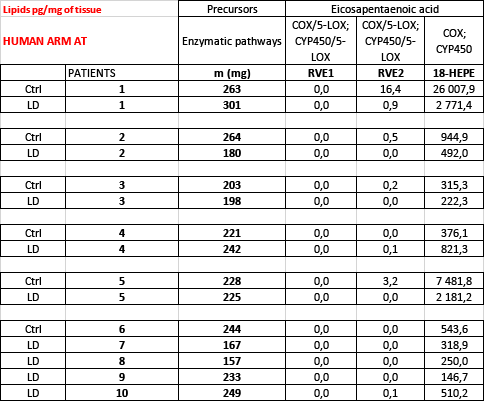


**Supplementary Table 6**: Dosage of EPA-derived lipids in human lymphedema adipose tissue biopsies. Source data are provided as a Source data file.


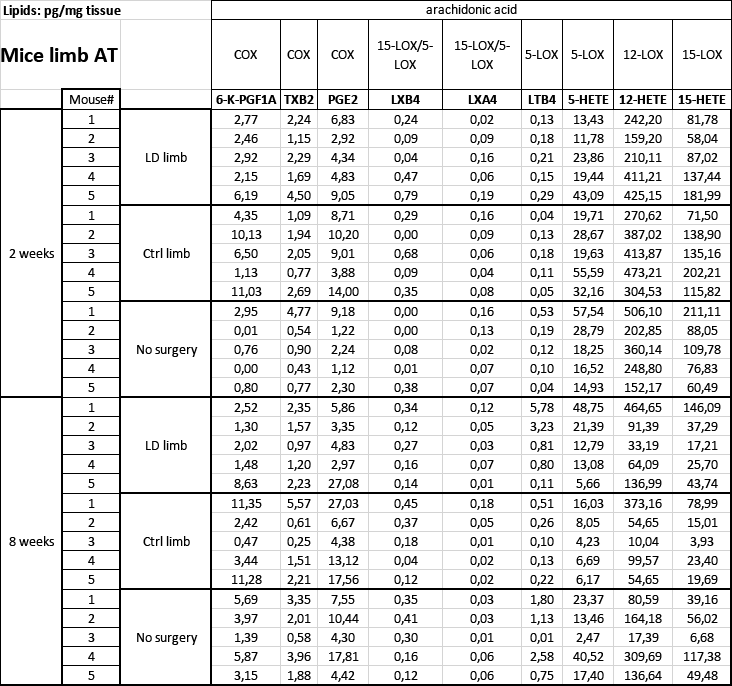


**Supplementary Table 7**: Dosage of AA-derived lipids in mice lymphedema adipose tissue biopsies 2 and 8 weeks after surgery. Source data are provided as a Source data file.


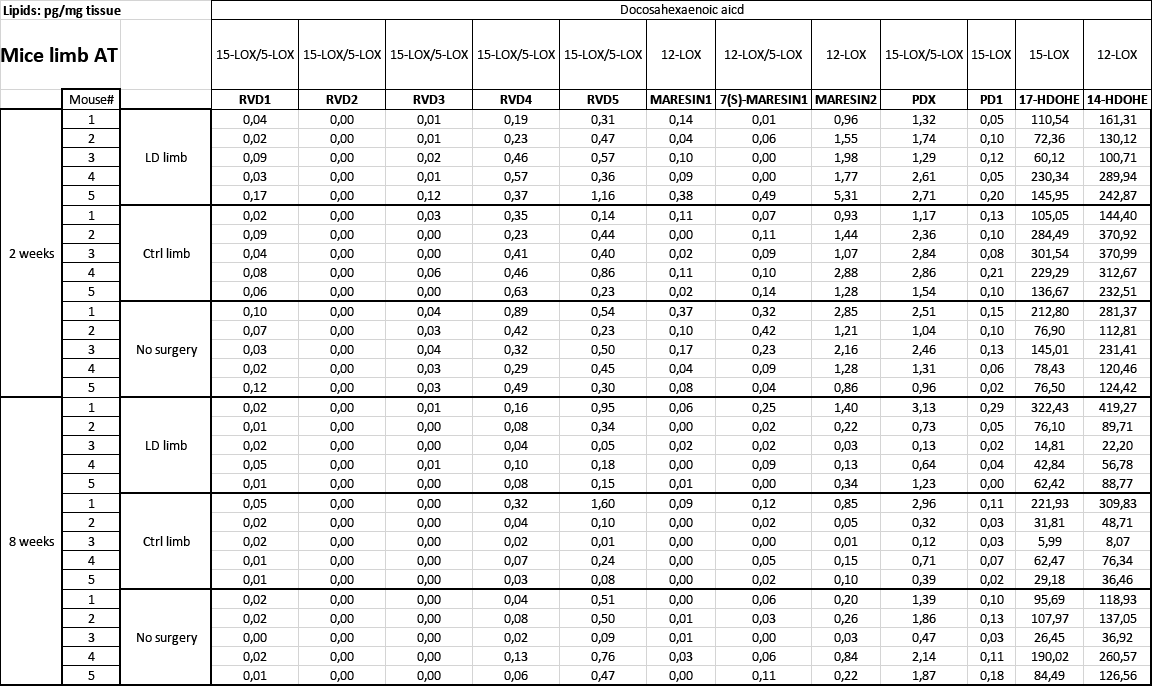


**Supplementary Table 8:** Dosage of DHA-derived lipids in mice lymphedema adipose tissue biopsies 2 and 8 weeks after surgery. Source data are provided as a Source data file.


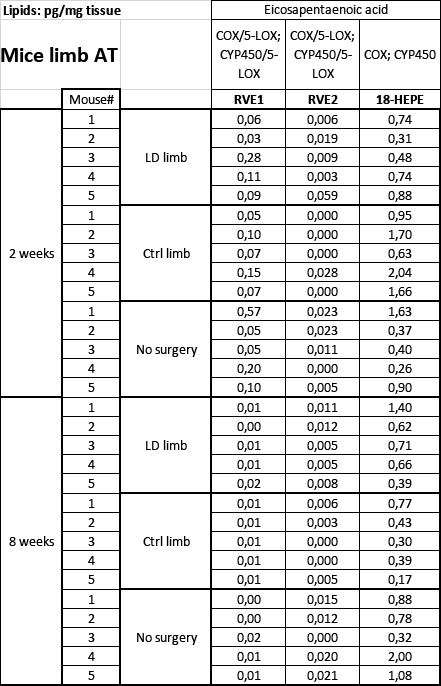


**Supplementary Table 9**: Dosage of EPA-derived lipids in mice lymphedema adipose tissue biopsies 2 and 8 weeks after surgery. Source data are provided as a Source data file.


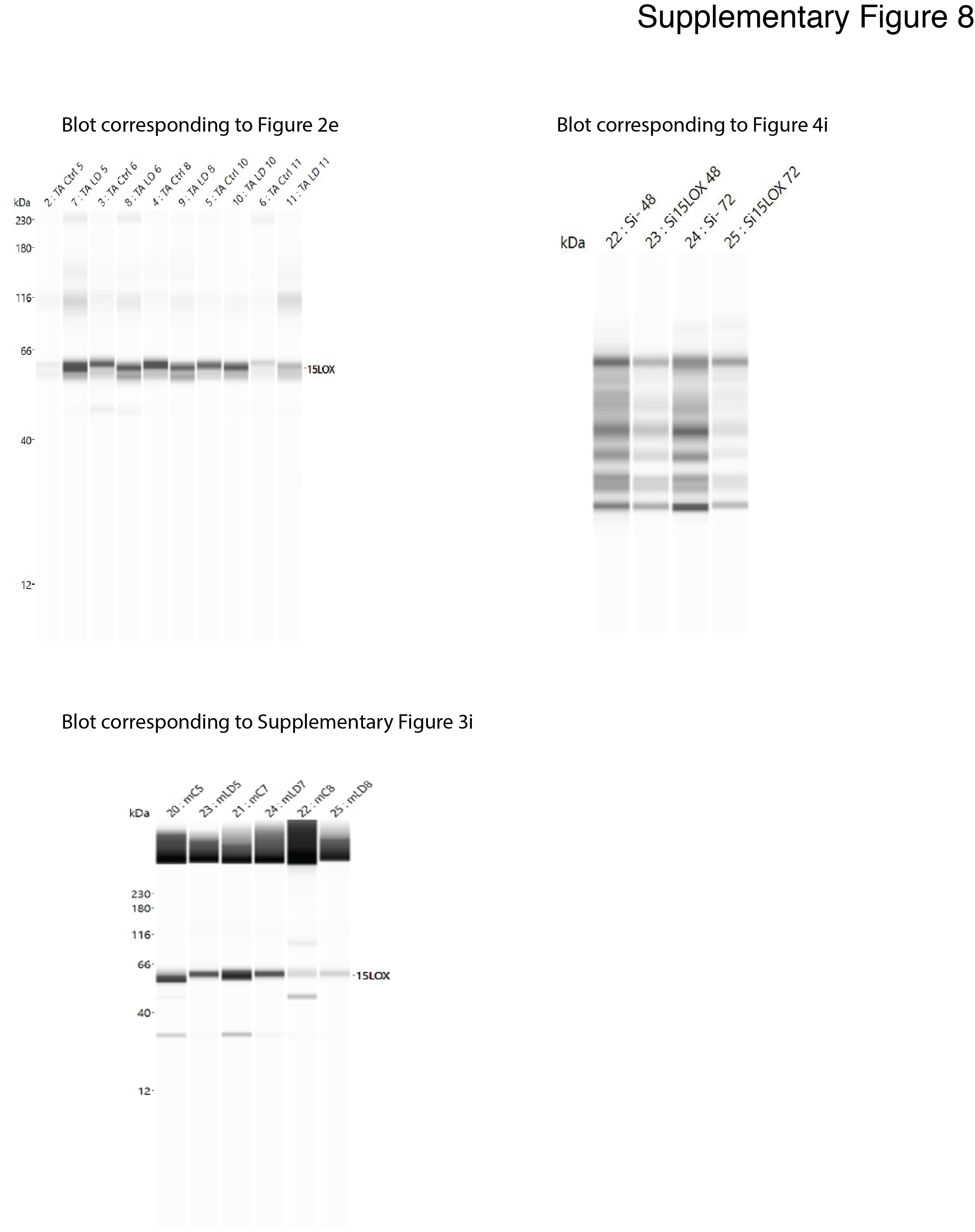


List of oligonucleotides

|  | Forward primers | Reverse primes |
| --- | --- | --- |
| 15-LO | 5’-AGCCCCAACTAATGACACGG-3’ | 5’- TGACCCCATCCTCACATTGC-3’ |
| CCL21 | 5’-CCTTGCCACACTCTTTCTCCC-3’ | 5’-CAAGGAAGAGGTGGGGTGTA-3’ |
| SPHK1 | 5’-CCGGTAGATGCACACCTTGT-3’ | 5’-TGGGTGCAGCAAACATCTCA-3’ |
| SPHK2 | 5’-CTAGATCGCCCTGACTGGGA-3’ | 5’-CTGGCTCAAATCCCCCGTG-3’ |
| S1PR1 | 5’-CCCCATGTGAAAGCGTCTCT-3’ | 5’-TGCACACACTCACTTGGGTT-3’ |
| LTBR | 5’-TGGAAGAGCCACCCTTCTCT-3’ | 5’-AGCAGTGGCTGTACCAAGTC-3’ |
| ICAM1 | 5’-CTCCAATGTGCCAGGCTTG-3’ | 5’-CAGTGGGAAAGTGCCATCCT-3’ |
| VCAM1 | 5’-TTCCCTAGAGATCCAGAAATCGAG-3’ | 5’-CCTGCAGCTTACAGTGACAGAGC-3’. |
| IFNa | 5’- ACCTCAGGAACAAGAGAGCC-3’ | 5’-CTTCTCCTGCGGGAATCCAA-3’ |
| IFNb | 5’- CCAGCTCCAAGAAAGGACGA-3' | 5’-TGGATGGCAAAGGCAGTGTA-3' |
| LTBR | 5’- GGCACCCAGAGGGAGAAGA-3’ | 5’-CTGCCAGGTCAGGGAAATGT-3’ |
